# Developmental validation of a 6-dye typing system with 27 loci and application in Han population of China

**DOI:** 10.1038/s41598-017-04548-1

**Published:** 2017-07-05

**Authors:** Yaju Liu, Lihong Guo, Haiying Jin, Zheng Li, Rufeng Bai, Meisen Shi, Shuhua Ma

**Affiliations:** 1 Institute of Criminal Sciences and Technology, Municipal Public Security Bureau of Xuchang, Xuchang, 461000 P.R. China; 2 Henan Provincal Public Security Department, Criminal Science and Technology Institute, Zhengzhou, 450003 P.R. China; 3 Health GeneTech, Ningbo, 315000 P.R. China; 4 Key Laboratory of Evidence Law and Forensic Science, Ministry of Education, Beijing, 100088 P.R. China; 5 Department of Radiology, First Affiliated Hospital, Medical College of Shantou University, Shantou, 515041 P.R. China

## Abstract

In this study, a novel 27-locus system (now known as the SureID PanGlobal system), including 24 autosomal STRs (D3S1358, TH01, D21S11, D18S51, Penta E, D12S391, D6S1043, D2S1338, D1S1656, D2S441, D5S818, D13S317, D7S820, D19S433, CSF1PO, Penta D, vWA, D8S1179, TPOX, FGA, D16S539, D22S1045, SE33, D10S1248), two Y-chromosome markers (DYS391 and Y-indel) and the sex determining marker, Amelogenin was developed with six fluorescent dyes labeling. The included STR loci belonged to the core loci in the Combined DNA Index System (CODIS) and the European Standard Set (ESS) as well as some additional loci commonly used in commercial kits and national DNA databases. This paper describes the validation studies conducted with the SureID PanGlobal system using Applied Biosystems 3500 XL Genetic Analyzer for fragment length detection that included the analysis of the following parameters and aspects: PCR conditions, sensitivity, species specificity, inhibition, precision, stutter, DNA mixtures, and stability studies with crime scene samples. The studies demonstrated that the SureID PanGlobal system is reproducible, accurate, sensitive and robust for forensic application and databasing. Additionally, the whole cycling time of the system can finish within 65 minutes, which was developed specifically for rapid and reliable generation of DNA profiles obtained from blood, buccal swabs and forensic stains.

## Introduction

Short tandem repeats (STRs) typing methods are widely used for paternity testing, human identification, and DNA database searches in criminal investigations^1, 2^. In light of the stringent demand for developing more efficient and discriminative typing systems following the Combined DNA Index System (CODIS) and the European Standard Set (ESS) recommendations^3^, a series of commercial STR kits incorporated 15–24 core markers into single multiplex systems have been produced with the development of new genetic analyzer platforms and manufacturing improvements^4–9^. PowerPlex Fusion kit of Promega Corporation^4^, AmpFlSTR GlobalFiler kit^7^ of Thermo Fisher Scientific, and Goldeneye 25A^8^ of Peoplespot Inc R&D (China) are main commercially available STR kits being used for forensic application in China.

However, as DNA databases continue to grow and international cooperation increases, there is still a need to develop sufficient multiplex systems to facilitate data sharing and to minimize adventitious matches. In this sense, we developed a 6-dye typing system (now known as the SureID PanGlobal Human DNA Identification Kit) consisting of 24 autosomal STRs which combined both CODIS and ESS core STR loci (CSF1PO, FGA, TH01, TPOX, vWA, D3S1358, D5S818, D7S820, D8S1179, D13S317, D16S539, D18S51, D21S11, D22S1045, D2S441, D1S1656, and D12S391), 7 other routinely tested loci (Penta D, Penta E, D2S1338, D19S433, D6S1043, D10S1248, and SE33) included to increase the discriminatory power of the multiplex, plus amelogenin (AMEL) for sex discrimination and DYS391, Yindel, the last loci incorporated to prevent improper gender determination when null Y alleles or deletions of the Y-chromosome may involve the locus amelogenin^10–12^. That means the SureID PanGlobal system can detect the genotyping results at all the STRs contained in the above three commercially available STR kits and can be compared with all the main international DNA databases. Furthermore, formulated with high performance Master Mix, the SureID PanGlobal system enables direct amplification of blood, buccal samples and forensic stains, and reduces the cycling time within 65 min.

Developmental validation studies of the SureID PanGlobal and forensic application in the Central Chinese Han population were explored. Tests were conducted for PCR conditions (including annealing temperatures and cycling numbers), sensitivity, mixture analysis, species specificity, performance on degraded DNA, performance with simulated inhibition, reproducibility-concordance tests, stutter analysis, accuracy, and precision. The conclusions reported here support the fact that the 6-dye SureID PanGlobal system is suitable for database applications and human identification in casework.

## Materials and Methods

### Ethics Statement

Human blood samples and tissue samples were collected upon approval of the Ethical committee of Medical College of Shantou University, China. Before getting involved in the study, all the participants signed the written informed consents for the human experiments and subsequent analyses. Animal blood samples were collected with the approval of the Animal Use Committee of Medical College of Shantou University, China. This study was approved by the Ethics Committee of Medical College of Shantou University, China.

### Marker selection and characterization

Selection of 27 candidate loci was considered as follows: (1) the core loci in CODIS and ESS, (2) available loci used in the main commercial kits in China: AmpFlSTR GlobalFiler kit of Thermo Fisher Scientific; PowerPlex Fusion kit of Promega Company; and Goldeneye 25 A of Peoplespot Inc R&D (China). Characterization of each locus (chromosomal location, physical position, repeat motif, genotype of control DNAs, and observed allele range) was listed in Table 1.

**Tablee 1:** General information of 27 Marker.

Marker	Chromosomal location	Basic repeat Motif	Note	Primer concentration (µM)	Amplicon size (bp)	Allele range	Control 9948	Control 9947	Dye label
D3S1358	3p21.31	TCTA /TCTG	US core locus, CODIS	0.84	107–147	12–20	15, 17	14, 15	6FAM
TH01	11p15.5	AATG/ATG	US core locus, CODIS	0.50	157–196	4–13.3	6, 9.3	8, 9.3	
D21S11	21q21.1	TCTA/TCTG	US core locus, CODIS	0.67	211–274	24–38	29, 30	30	
D18S51	18q21.33	AGAA	US core locus, CODIS	0.66	285–366	8–27	15, 18	15, 19	
Penta E	15q26.2	AAAGA	Commercially available from Promega	0.67	373–491	5–24	11, 11	12, 13	
Yindel	Yq11.221	TTCTC	Commercially available from GlobalFiler	1.34	99–104	1, 2	2	–	HEX
DYS391	Yq11.21	TCTA	European core Y–STR; SWGDAM recommended	0.54	115–159	6–16	10	—	
D12S391	12p12	AGAT/AGAC Complex	European recommended locus	1.26	169–221	14–26	18, 24	18, 20	
D6S1043	6q15	AGAT	Specially selected for Chinese population	0.30	242–310	8–21	12, 12	12, 18	
D2S1338	2q35	TGCC/TTCC	European locus	0.70	328–389	15–27	23, 23	19, 23	
D1S1656	1q42	TAGA	European recommended locus	1.36	394–441	11–18.3	14, 17	18.3	
D5S818	5q23.2	AGAT	US core locus, CODIS	0.50	114–155	7–16	11, 13	11	TAMRA
D13S317	13q31.1	TATC	US core locus, CODIS	0.60	170–206	7–15	11, 11	11	
D7S820	7q21.11	GATA	US core locus, CODIS	0.65	226–262	6–15	11, 11	10, 11	
D19S433	19q12	AAGG	US core locus	1.30	282–332	10–17.2	13, 14	14, 15	
CSF1PO	5q33.1	AGAT	US core locus, CODIS	1.20	337–378	6–15	10, 11	10, 12	
Penta D	21q22.3	AAAGA	Commercially available from Promega	1.30	394–465	2.2–17	8, 12	12	
D2S441	2p14	TCTA	European recommended locus	0.65	82–118	8–17	11, 12	10, 14	ROX
vWA	12p13.31	TCTA /TCTG	US core locus, CODIS	4.60	124–189	10–24	17, 17	17, 18	
D8S1179	8q24.13	TCTA /TCTG	US core locus, CODIS	1.80	206–255	7–18	12, 13	13	
TPOX	2p25.3	AATG	US core locus, CODIS	2.70	265–308	6-13	8, 9	8	
FGA	4q28	CTTT	US core locus, CODIS	1.90	314–454	16–46.2	24, 26	23, 24	
AmelogeninX/Y	p22.1–22.3/p11.2	–/AAAGTG	Sex-Typing Marker	1.50	110–116	X, Y	X, Y	X	ALEXA594
D16S539	16q24.1	GATA	US core locus, CODIS	2.00	123–163	5–15	11, 11	11, 12	
D22S1045	22q12.3	ATT	European recommended locus	2.10	172–211	7–19	16, 18	11, 14	
SE33	6q14	Complex AAAG	German Core Loci	2.30	236–368	4.2–36	23.2, 26.2	19, 29.2	
D10S1248	10q26.3	GGAA	European recommended locus	2.50	387–431	8–18	12, 15	13, 15	

### Primer, internal size standard, matrix standard set and allelic ladders

PCR primers were designed for amplicons smaller than 500 bp using the Primer3 program (http://bioinfo.ut.ee/ primer3-0.4.0/primer3/input.htm). Each primer was checked for potential structures of the self dimmer using the AutoDimer v1.1 software and non-specific hybridizations in other genome regions using NCBI Basic Local Alignment Search Tool (BLAST). 27 loci were organized by expected amplicon size and assigned to five dye-labeling fluorochromes (6FAM, HEX, TAMRA, ROX, or ALEXA594) in order to achieve an evenly balanced genotyping assay for a single PCR and electrophoretic separation. The internal size standard labeled with “Orange” dye color was selected from the SIZE 500 (Health Gene Tech Co, China) for calculating the fragment sizes of PCR products. It contains 18 dye-labeled DNA fragments with the length of base pairs of: 75, 100, 125, 150, 175, 200, 225, 250, 275, 300, 325, 350, 375, 400, 425, 450, 475, and 500. Spectral calibration with dye matrix is essential for the data analysis. We chose and labeled six amplified fragments from pUC18 DNA: 100 bp (Orange), 125 bp (ROX), 140 bp (TAMRA), 150 bp (HEX), 160 bp (FAM), and 165 bp (ALEXA594).

Allelic ladders were created with 27-locus multiplex using a combination of individual templates that represent the range of alleles observed in the studied populations. The successful clones of each allele were diluted, mixed, analyzed and balanced to produce a single allelic ladder for each STR. Those single allelic ladders were mixed to combine a “cocktail”in appropriate portions. All alleles included in the in-house ladder were then sequenced to confirm the number of repeats using Big Dye Terminator v.3.1 chemistry (Thermo Fisher Scientific). When the in-house ladder was confirmed and optimized, panel and bin files for the GeneMapper ID-X Software V1.4 were programmed.

### DNA amplification and 3500 XL detection

Two PCR amplification reactions were tested in this study following the PCR setup proportions according to the SureID PanGlobal system user guide. A PCR with a final volume of 25 µL, used for the vast majority of validation studies reported here, was prepared by combining 12.5 µL PCR Master Mix, 6.25 µL Primer Mix, to which 6.25 µL of 0.5~4 ng of template DNA and DNase/RNase-Free H_2_O was added. A PCR with a final volume of 10 µL was also tested for reference and control samples by combining 5.0 µL PCR Master Mix, with 2.5 µL of Primer Mix, and 2.5 µL of of 0.5~4 ng of template DNA and DNase/RNase-Free H_2_O. The PCR Master mix included DMSO 10 mM, Tris buffer 125 mM, KCL125mM NH_4_NO_3_ 65 mM, dNTP_S_ 7.5 mM and bovine serum albumin (BSA) 2.5 mg/mL. The Primer mix included appropriate concentration of primers, 7.5 mM MgCl_2_, and 2-4U/6.25 µL High Specificity *Taq* DNA polymerase (Dongsheng BioTech, China). Amplification was performed on a GeneAmp 9700 thermal cyclers (Life Technologies). Standard thermal cycling conditions consisted of enzyme activation at 95 °C for 5 min, 28~30 cycles according to DNA sample types at 94 °C for 10 sec, annealing at 61 °C for 1 min, extension at 70 °C for 30 sec. A final extension step was performed at 60  °C for 15~30 min, followed by a final hold at 4 °C.

PCR products were prepared by combining 1 µL of each amplified product with 9 µL in a 17:1 mixture of Hi-Di formamide (Life Technologies) and SIZE 500(Health Gene Tech Co, China). Capillary electrophoresis was performed with ABI Prism 3500 Genetic Analyzer (Life Technologies) to process the data from the six dyes 6-FAM (blue), HEX (green), TAMRA (yellow), ROX (red), ALEXA594(Purple) and SIZE-500(orange) after an appropriate matrix. Standard run conditions involved the following parameters: sample injection for 15 sec at 1.2 kV and electrophoresis at 15 kV for 1210 sec in POP-4 polymer with a run temperature of 60 °C as indicated in the HID36_POP4 xl run module. All genotyping data was analyzed using GeneMapper ID-X Software V1.4 (Life Technologies) with the analysis settings specified in the SureID PanGlobal system (panels, bins, and stutter files).

### Developmental Validation studies

Developmental Validation studies were performed in accordance with the validation guidelines issued by the Scientific Working Group on DNA Analysis Methods (SWGDAM) (http://swgdam.org/SWGDAM_Validation_Guidelines_APPROVED_June_2015.pdf) and the DNA Working Group of the European Network of Forensic Science Institutes (http://www.enfsi.eu/sites/default/files/docu-ments/minimum_validation_guidelines_in_dna_Profiling_v2010_0.pdf).

A serial dilutions of control DNA 9948 with quantities from 2 ng to 31.25 pg per reaction as the template were analyzed in triplicate to evaluate the sensitivity.

For the repeatability and reproducibility study, 20 prepared genomic DNA samples with known STR profiles were evaluated in two accredited laboratories in quintet.

Artificially degraded control DNA 9948 was sheared using adaptive focused acoustics (Covaris system, Covaris lnc, USA) to generate average fragment lengths of 500 bp, 300 bp, or 150 bp to test the performance on degraded DNA.

A mixed male/female DNA sample with known ratios (9947 A and 9948 mixed at 1:10, 1:8, 1:4, 1:1, 4:1, 8:1 and 10:1) for a total of 1 ng of template DNA were prepared for the mixture study.

Species specificity studies were conducted using whole blood samples from primate mammal (macaque), non-primate mammals (dog, horse, mouse, pig, sheep, chicken, cow, rabbit, and fish) and several human-associated microbial species (*Escherichia coli, Micrococcus luteus, and Streptococcus salivarius* at a DNA input of 4–10 ng.

Stability studies were performed using several common forensic inhibitors purchased from Sigma–Aldrich Corporation (USA): hematin, humic acid, tannic acid and calcium. Inhibitor concentrations ranged here were: 50 µM, 75 µM, 100 µM, 150 µM and 200 µM of hematin, 20 ng/µL, 50 ng/µL, 70 ng/µL and 90 ng/µL of humic acid, 50 ng/µL, 100 ng/µL, 150 ng/µL, and 200 ng/µL of tannic acid, and 0.2 mM, 0.4 mM, 0.8 mM, 1.0 mM, and 2.0 mM of calcium. All inhibitors were previously prepared and then added to 0.5 ng 9948 DNA prior to PCR.

Accuracy, balance, stutter, and concordance studies were performed using DNA samples from blood or saliva swabs of 457 Han nationality samples, which were previously analyzed using the PowerPlex Fusion (Promega Corporation), GlobalFiler (Life Technologies), and Goldeneye 25 A (Peoplespot INC R&D (China) kits. SureID PanGlobal Allelic ladder was performed for size precision studies. Allelic ladder was run on two different instruments because of type and concentration of polymer mixture, run temperature and electrophoresis conditions. Data from a total of 457 allelic ladder samples was collected including 240 samples (three successive runs) on the 3500 Genetic Analyzer and 217 samples (three successive runs) on the 3500 XL Genetic Analyzer. The mean value of size and standard deviation (SD) was calculated for each allelic ladder allele on each instrument.

Thirty-eight crime scene samples were used in the stability studies. They included 15 bloodstains, 5 semen stains, 5 saliva stains, 5 muscle samples, 5 bone samples, and 3 formalin fixed and paraffin embedded biopsies (FFPEB). All crime scene samples were previously quantified using Quantifiler Duo Human DNA quantification Kit (Thermo Fisher, USA) and analyzed with PowerPlex Fusion, GlobalFiler, and Goldeneye 25 A kits. Combined loci in these different commercial kits can obtain 27 loci in the SureID PanGlobal system.

To collect more allelic frequencies at all loci and to estimate the forensic efficiency of the SureID PanGlobal system, an extensive population study including 1136 individuals (776 males and 360 females) of Han participated was conducted.

### Statistical analysis

Evaluation of Hardy–Weinberg equilibrium (HWE), linkage disequilibrium (LD) among loci pairs were performed with the software Arlequin 3.5 (http://cmpg.unibe.ch/software/arlequin3.5). Allelic frequencies and forensic parameters including power of discrimination (DP), polymorphism information content (PIC), probability of paternity exclusion in duos (PEduo) and probability of paternity exclusion in trios (PEtri) were calculated using the Power Markerv3.25 software13. For the Y-STR locus DYS391, allelic frequencies of the studied population were calculated by direct counting method; gene diversity (GD) was calculated using the formula GD = n (1 − ∑Pi^2^)/(n − 1), where *Pi* is the frequency of the ith allele and n indicates the total number of samples.

### Quality control

The main experiments were carried out at the Key Laboratory of Evidence Law and Forensic Science, Ministry of Education, P.R.China, which is an accredited laboratory (ISO 17025), in accordance with quality control measures. All the methods were carried out in accordance with the approved guidelines of the Key Laboratory of Evidence Law and Forensic Science, Ministry of Education, P.R.China.

## Results and Discussion

Ten STR loci (D3S1358, TH01, DYS391,D12S391, D5S818, D13S317, vWA, D2S441, D16S539, and D22S1045) were designed within 220 bp for maximized performance on partially degraded samples. All primers were tested initially in previously developed multiplexes, first as singleplexes and then in a multiplex system. The reaction elements of the PCR system (Reaction Mix) were already optimized and fixed during our previous work (data not shown). Based on the results of genotyping profiles, the optimization of each primer’s concentrations in the final Primer mix was performed. Primer-related information on each locus (primer concentration, PCR product size, and dye label) was listed in Table 1.

Annealing temperatures between 56 °C and 66.5 °C were tested in triplicates with 1.0 ng Control DNA 9948. Full profiles with balanced inter-locus signals were obtained when PCR was performed with annealing temperatures between 59 °C and 63.5 °C. The best amplification profile was obtained at the annealing temperature of 61 °C (Supplementary Fig. 1). Both 10 µL reaction volume and 25 µL reaction volume showed the same results. Results also demonstrated reliable, cost-effective performance from 10 µL reaction volume that showed even higher peak height intensity than the standard 25 µL PCR volume due to a higher concentration of PCR products in the reduced reaction volume.

**Figure 1:**
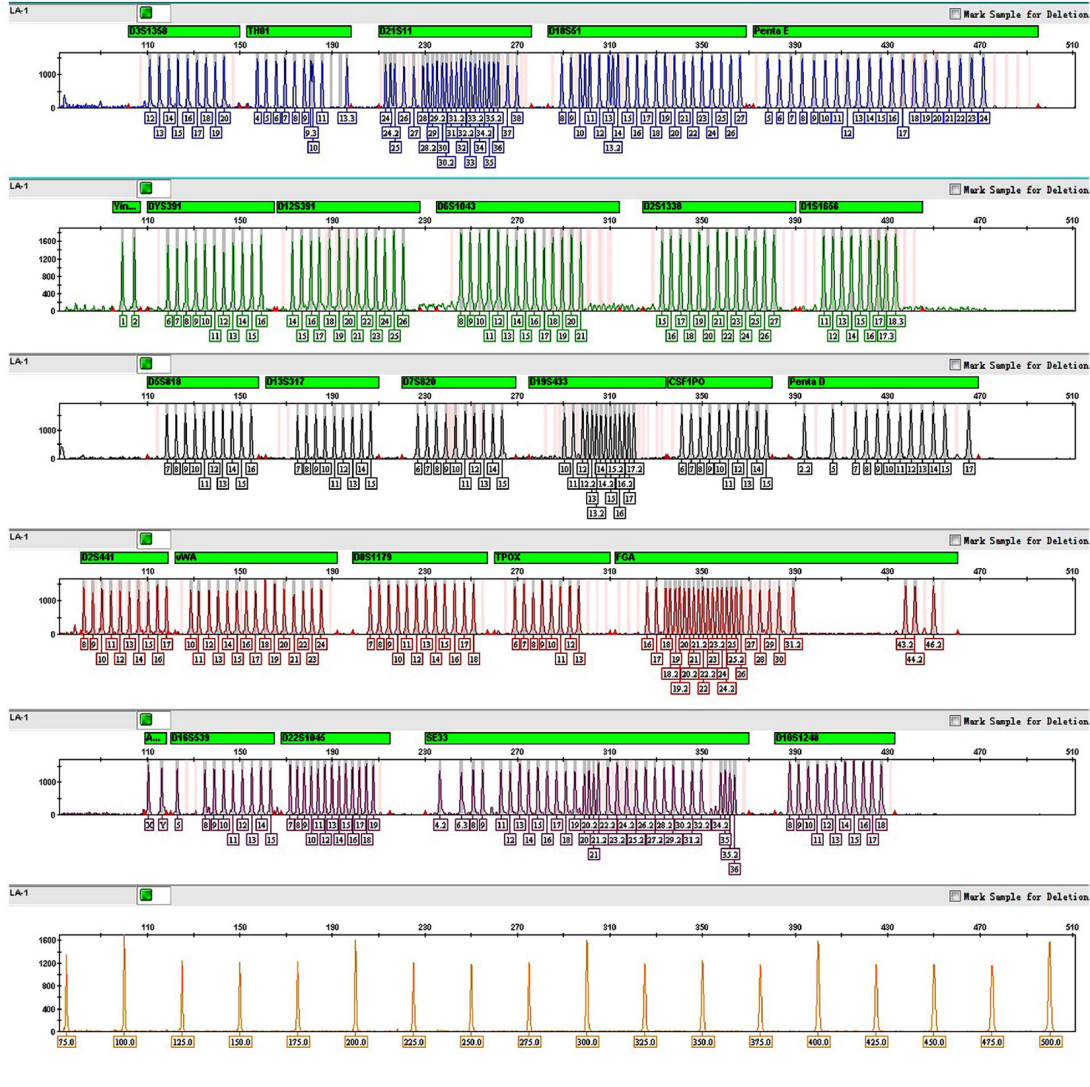
Electropherogram of allelic ladders and internal size standard in the SureID PanGlobal system. The five dye panels for allelic ladders correspond to (from top to bottom) 6FAM (blue), HEX (green), TAMRA (yellow), ROX (red),and ALEXA594(purple) dye-labeled peaks. The sixth panel reserved for internal size standard labels an orange SIZE500 dye. The genotype is shown with the allele number displayed underneath each peak. One microliter each of allelic ladders and internal size standard were simultaneously analyzed on an Applied Biosystems 3500 XL Genetic Analyzer with a 1.2 kV, 15 s injection.

Altering the number of PCR cycles can be used to optimize the reaction conditions for various DNA template concentrations. Cycle numbers can be either increased to enhance amplification signals when working with low-copy-number DNA, or decreased to speed up the protocol when there is abundant DNA in the sample. Cycle numbers were increased to 32 (or 34 for reactions containing 125, 62.5, 31.25, 15, or 8 pg Positive Control 9948). As expected, the signal intensities of the amplified products increase with higher cycle numbers. However, it should be noted that cycle number of more than 32 may not necessarily result in more called alleles from the low-template-DNA sample. Furthermore, because of stochastic effects, increased peak imbalances, non-specific PCR products or dropouts may in general be observed for low-copy-number samples (100 pg or less template DNA) regardless of cycle numbers. In this experiment, using a threshold of 100 RFU for allele calling, the number of allelic dropouts due to stochastic effects was not significantly reduced when more PCR cycles were applied (Supplementary Fig. 2). Overall, the final optimized PCR conditions: an initial denaturation step of 5 min at 95 °C, 28~30 cycles according to DNA sample types at 94 °C for 10 sec, 61 °C for 1 min and 70 °C for 30 sec, with a final extension step performed at 60 °C for 15~30 min. The whole PCR reaction can finish within 65 min, which was developed specifically for rapid and reliable generation of DNA profiles obtained from blood, buccal swabs and forensic stains. After the development and optimization of the multiplex, 27 loci were successfully amplified in a single PCR reaction. Allelic ladders and internal size standard of SureID PanGlobal system were shown in Fig. 1. The profile generated by using 1.0 ng Control DNA 9948 was attached in Supplementary Fig. 3.

**Figure 2:**
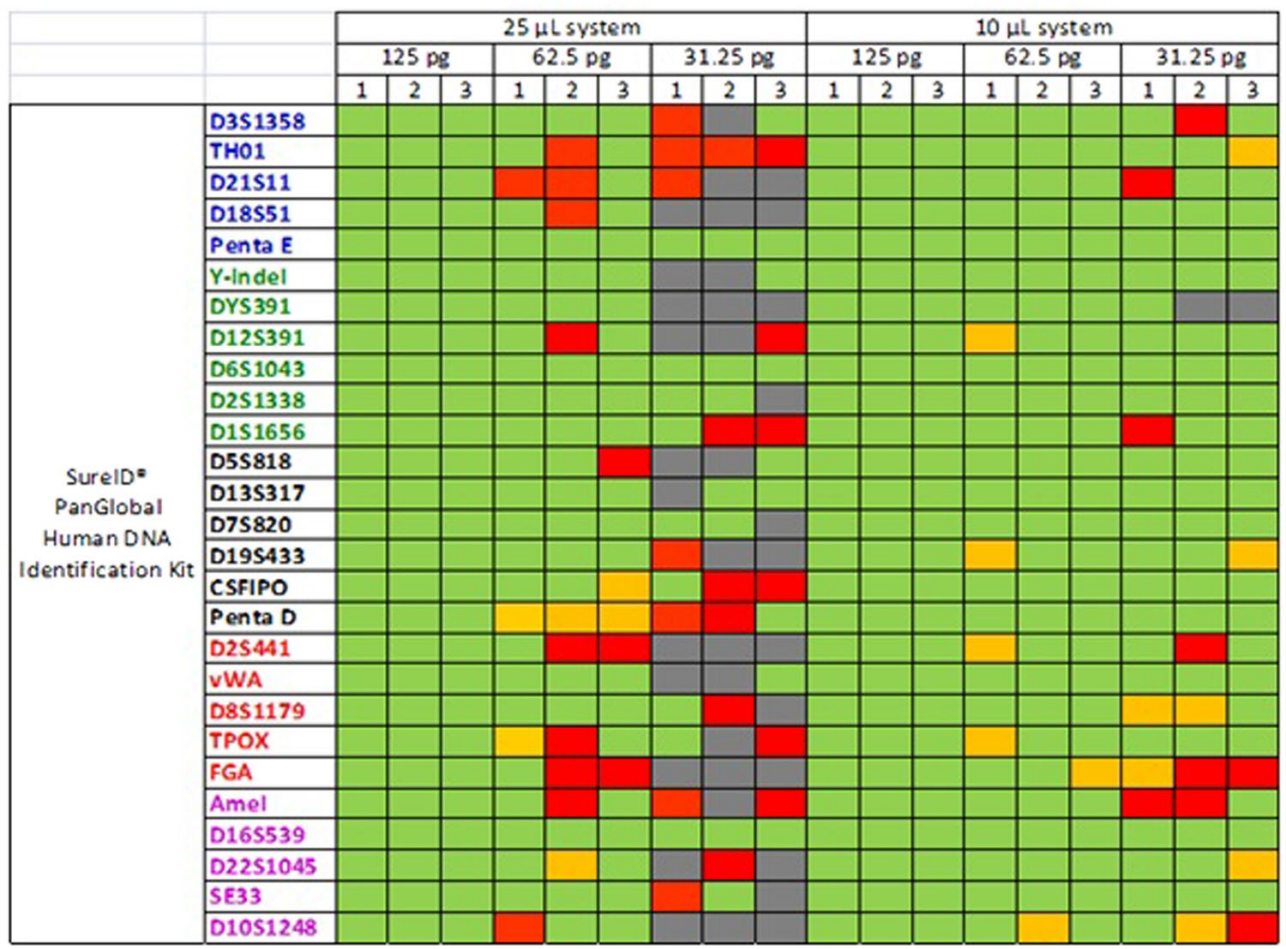
The sensitivity of the 25 µL system and the 10 µL system. 100 RFU was used as a threshold for detection. Green represents peak height ratio >50% within the same loci. Yellow represents peak height ratio <50% within the same loci. Red represents one of alleles drop-out within the same loci. Gray represents both of alleles drop-out within the same loci.

For the sensitivity test, control DNA 9948 was serially diluted from 2 ng to 31.25 pg per reaction as the template in triplicates. Full profiles were consistently obtained at 125 pg per reaction using the standard conditions. In the 10 µL reaction system, occasional allele dropouts were found when ≤31.25 pg DNA was used, whereas in the 25 µL reaction system, occasional allele dropouts were found when ≤62.5 pg DNA was used as template (Fig. 2). As expected, the number of dropouts increases with decreasing DNA concentration. The recommended amount of input DNA is 1.0~4.0 ng per reaction for yielding high quality STR profiles. Excessive amount of template DNA will lead to imbalanced amplification of alleles and nonspecific peaks, while insufficient template DNA will cause allelic loss, or failure of amplification.

For the repeatability and reproducibility test, full concordant allele results were obtained among the DNA profiles obtained by two laboratories in the analysis of 5 replicates of 20 human genomic DNA samples with known STR profiles using 1.0 ng of genomic DNA input, demonstrating the reproducibility and the repeatability of the SureID PanGlobal system.

Human DNA extracted from evidence samples exposed to adverse environmental conditions may show various degrees of degradation. To determine the efficiency of amplification with degraded samples, artificially degraded Control DNA 9948 was tested by shearing average fragment lengths of 500 bp, 300 bp, or 150 bp. Fragment lengths were verified on an agarose gel and 0.5 ng DNA was used as the template for amplification (all samples in duplicate). Full profiles were obtained with an average DNA length of 500 bp. The results show that the larger the amplicon sizes, the more severe of signal drop off as DNA degradation progresses (Supplementary Fig. 4). Increased amounts of template can be used to improve results for heavily degraded DNA.

For the correct interpretation of results from DNA mixtures, it is important to know the limit of the minor contributing components that can be resolved. Samples were created by mixing control DNA 9947 and 9948 in ratios of 1:10, 1:8, 1:4, 1:1, 4:1, 8:1 and 10:1 for a total of 1 ng of template DNA. The limit of detection of the minor components was determined by analyzing non-overlapping alleles of both types of DNA. All expected alleles were found for minor components of 1:4 and 1:8 mixtures. As the mixture ratios became higher there was a decrease in the percentage of minor alleles that could be identified. 10:1 and 1:10 typically resulted in partial profiles of the minor components. An example for 1:10 and 10:1 mixtures was shown in Supplementary Fig. 5. Since both mixtures contain ≤100 pg of the minor component, the results were in concordance with the sensitivity for single-source samples reported here. In order to increase the sensitivity for the minor components, higher overall DNA amounts may be used if the amount of available DNA is not limited.

Non-human DNA can be present in forensic casework samples. It is critical that assays show no cross-reactivity between species. To verify species specificity of the SureID PanGlobal system, DNA samples from other species were separately tested following the standard assay protocol. Common pets, farm animals and some microbial DNA isolates were tested for species specificity. Negative results were obtained over a 50 RFU threshold except for Macaque and Horse. Macaque DNA produced an Amelogenin X peak, Penta E 6/7 peaks, and further off-ladder peaks in the FGA and D22S1045 loci. Horse DNA showed a low-level off-ladder peak in the purple panel at 107.58 bp. The detection of genetic information from non-human samples may help define the limits of the assay.

The stability to obtain profiles from DNA subjected to environmental PCR-inhibitors was evaluated. Typical environmental and purification-related PCR-inhibitors, humic acid, hematin, tannic acid, and calcium, were titrated into SureID PanGlobal reactions containing 0.5 ng Control DNA9948. With humic acid, full profiles were generated with ≤70 ng/µL (Supplementary Fig. S6-1). Full, concordant profiles were obtained with hematin concentrations ≤100 µM (Supplementary Fig. S6-2). Full profiles were generated with 100 ng/µL tannic acid (Supplementary Fig. S6-3). Lastly, full profiles were obtained with ≤1.0 mM calcium (Supplementary Fig. S6-4). Results demonstrated the SureID PanGlobal system could tolerate considerable concentrations of inhibitors.

**Figure 3:**
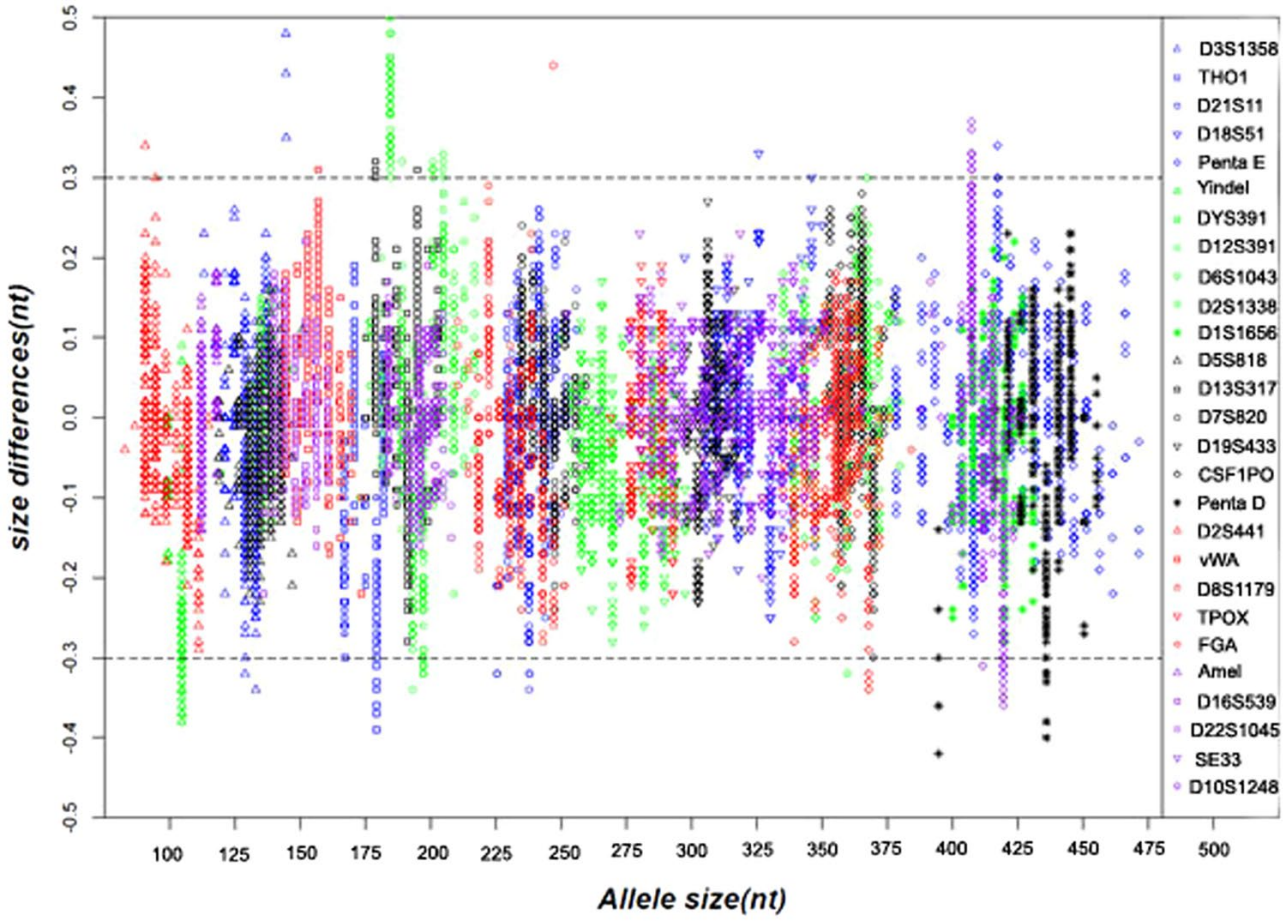
Size accuracy of the SureID PanGlobal system on Applied Biosystems 3500 XL Genetic Analyzer. Data points were collected from a total of 22815 alleles from 457 samples. The X-axis represents the nominal nucleotide sizes for the SureID PanGlobal allelic ladder mix. The dashed lines parallel to the X-axis represent the ±0.3 nt windows. The Y-axis represents the deviation of each sample allele size from the corresponding size of allelic ladder. All sample alleles are within ±0.5 nt from a corresponding allele in the allelic ladder mix.

For the accuracy study, 457 sample alleles were analyzed on Applied Biosystems 3500/3500 XL Genetic Analyzers with 36 cm array and POP-4 polymer. After confirming the accurate sequence of each allele in each sample, the average fragment size and standard deviation for each allele were calculated [Fig. 3]. The precision of size calling is crucial for accurate and reliable genotyping. Precision was measured by calculating the standard deviation in the size values obtained for an allele that is run in several injections on genetic analyzer. Supplementary Fig. 7 showed typical precision results obtained from multiple runs of the SureID PanGlobal allelic ladder mix on 3500/3500 XL Genetic Analyzers with POP-4 polymer using the SIZE-500. The highest Standard deviation (SD) was 0.064 nt in one set of injections for Penta E allele 20 on 3500 and 0.071 nt for Penta D allele 14 on 3500 XL. Overall, the SDs of all alleles on two instruments fell well below 0.15 nt, which ensured that sample allele was rarely sized outside of the ± 0.5 nt window. All sample alleles were sized within ± 0.5 bp from a corresponding allele in the allelic ladder mix which ensured the SureID PanGlobal system could reliably determine the genotypes and accurately detect the microvariant alleles that differ by a single nucleotide from complete repeat alleles. Furthermore, the recommended PCR performance criterion includes intra-locus balance >70%,intra-color balance >50%, and inter-color balance >30%^13, 14^. The balance calculations from 457 samples showed the mean values could conformed to the criteria: 85.88–96.90% intra-locus, 61.96–71.28% intra-color, 32.86% inter-color balances for DNA templates ranging from 4 ng to 0.5 ng.

Stutter is a well-characterized PCR artifact that refers to the appearance of a minor peak that is one repeat unit smaller (or less frequently, one repeat unit larger) than the major STR product^14^. The proportion of the stutter product relative to the main allele (stutter percentage)was measured by dividing the height of the stutter peak by the height of the main allele peak. The stutter ratio mean and standard deviations for each locus was calculated from a subset of 457 population samples with peak heights between 150 RFUs and 6000 RFUs (Supplementary Table 1). The estimated stutter filter for the GeneMapper ID and ID-X files (calculated as the mean stutter for the locus plus three standard deviations) was also displayed. The percent stutter is usually more pronounced for shorter repeat motifs and generally increases with allele length^15^. In our study the trinucleotide-repeat locus D22S1045 and the locus D12S391, which contains complex tetranucleotide repeats, displayed the highest relative stutter peaks with stutter filter heights at 20.14% and 22.82% of the main allele, respectively.

A full genotype concordance was achieved after the comparison of SureID PanGlobal profiling data obtained from 457 human genomic DNA samples with the profiling data obtained previously from the same set of samples with different commercial STR kits (GlobalFiler, PowerPlex Fusion, Goldeneye 25 A). Gender determination among the sex-markers (DYS391, Y Indel and AMEL) was also demonstrated concordant in all reference samples. Above results ensure that profiles generated with the SureID PanGlobal system are reliable and suitable for application.

**Figure 4:**
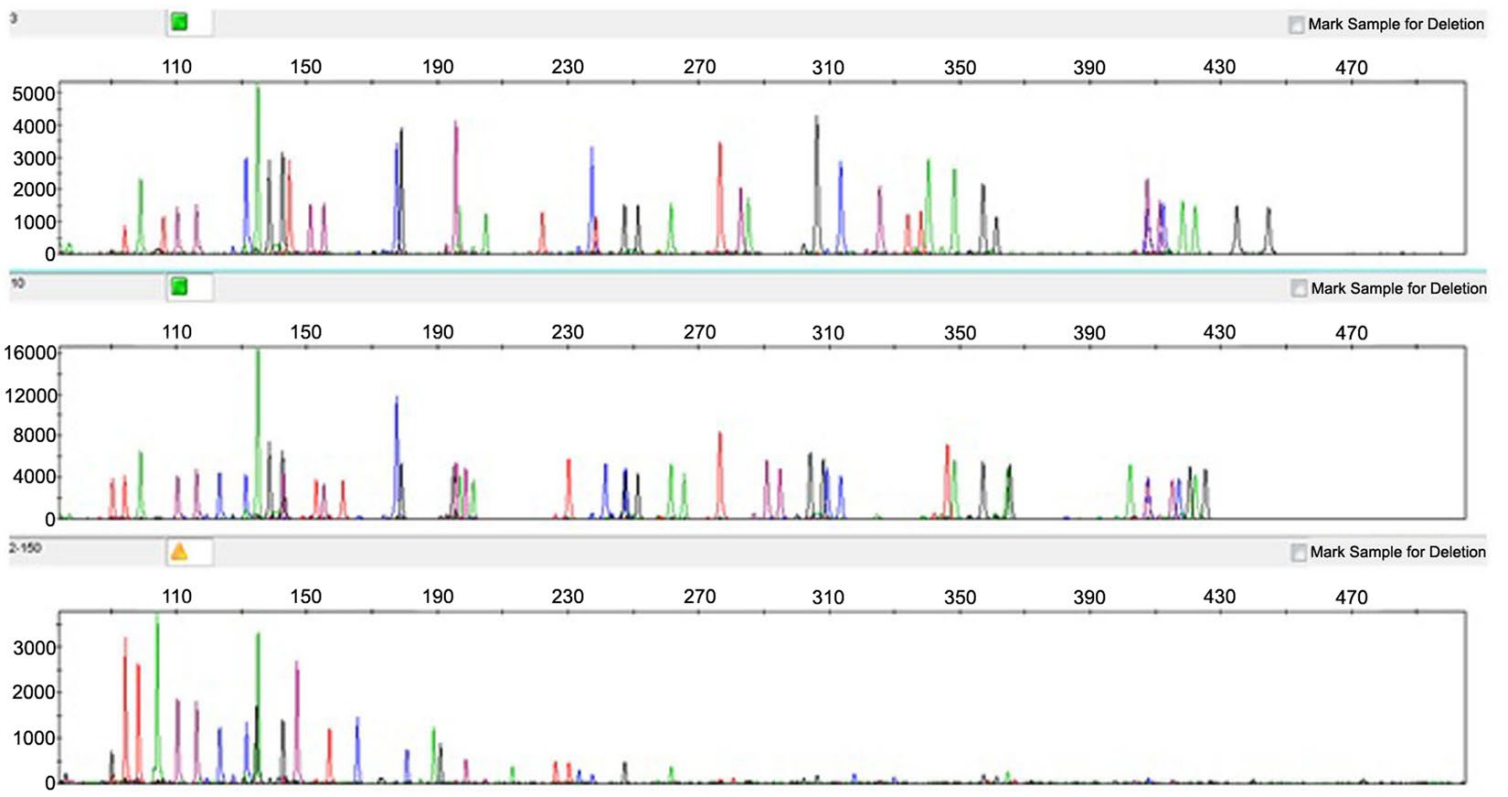
SureID PanGlobal electropherograms obtained from two different moderately degraded DNA bone samples (upper & medium panels) and from a severely degraded FFPEB sample (lower panel).

The SureID PanGlobal system was also tested with DNA extracts obtained from crime scene samples such as blood, saliva, semen stains, and from different tissues (including muscle, old bone samples, and formalin fixed and paraffin embedded biopsies) to evaluate the ability to obtain full or partial profiles. The SureID PanGlobal profiling results obtained from 25 biological stains showed full allele concordance with previously results genotyped by GlobalFiler, PowerPlex Fusion, and Goldeneye 25 A kits (data not shown). Degraded DNA obtained from 5 muscle samples and 5 bone samples (post-mortem interval from 6 month to 5 years) and from 3 severely degraded DNA sample retrieved from a formalin-fixed and paraffin-embedded biopsies were used to challenge the SureID PanGlobal against DNA degradation in parallel with the above three commercial STR kits. Six degraded DNA samples (reduced to the loci with allele sizes under 250 bp) rendered both full profiles or very informative partial STR profiles with both SureID PanGlobal and GlobalFiler kits. SureID PanGlobal full profiling offered in those samples the increased discriminatory power (over the GlobalFiler kit) to incorporate 3 additional autosomal STR loci (Penta D, Penta E, and D6S1043). For two severely degraded DNA samples (FFPEB) partial STR profiles (reduced to the loci with allele sizes under 150 bp) were obtained with the SureID PanGlobal and other 3 commercial kits. Figure 4 shows a full SureID PanGlobal profile electropherogram obtained from one moderately degraded DNA bone sample and a very partial profile obtained from a severely degraded FFPEB sample.

Population studies of the SureID PanGlobal system were performed using 1136 unrelated samples (360 females and 776 males) from Han nation of China. All studied 24 autosomal STRs of the system followed Hardy–Weinberg equilibrium. Allele frequencies and forensic parameters for the 24 STR loci derived from the population were shown in Supplementary Table 2. A total of 347 alleles with 1438 genotypes among the 1136 Central Han Chinese were detected for the 24 autosomal STR loci while SE33 had the greatest number of variants with 45 alleles. All autosomal STR loci showed high forensic efficiency with DP values above 0.8. Since no linkage disequilibrium was truly established after Bonferroni correction, the combined forensic efficiency parameters were calculated based on allelic frequencies while cumulative probability of paternity exclusion (PE) in trios (CPE_trio_) was 0.999999999973669, and cumulative probability of paternity exclusion in duos (CPE_duo_) was 0.999999701532747. These results suggest that the 24 autosomal STR loci included in this multiplex assay are highly polymorphic and informative for population genetics research and for individual identification and paternity testing in the Han population of China. For the Y-STR locus of DYS391 among the 776 Central Han males, 6 alleles were detected with GD value of 0.4578.

## Conclusions

In this paper, the development of SureID PanGlobal system and the application information in Han population of China are reported. With the development of new genetic analyzer platforms and manufacturing improvements, we recruited additionally purple color of fluorescent dye and two Y chromosome markers (DYS391,Y-Indel) in the system. D6S1043 was also chosen to incorporate into the system being the unique locus for Chinese. Ten loci (D3S1358, TH01, DYS391, D12S391, D5S818, D13S317, vWA, D2S441, D16S539, and D22S1045) were designed within 220 bp in length for maximized performance on degraded casework samples. 27 common and informative loci used throughout the world were included in this 6-dye typing multiplex. The most abundant number and degree of polymorphism of STRs included in the SureID PanGlobal system yields the highest power of discrimination currently obtainable from commercial kits according to our obtained data. In addition, the SureID PanGlobal system is developed specifically for rapid and reliable generation of DNA profiles obtained from blood, buccal swabs and forensic stains within 65-min cycling time.

As documented in SWGDAM and ENFSI DNA WG guidelines, minimal cross-reactivity, low-level sensitivity and mixture detection, precise and accurate allele calls, and robust performance with casework samples and in the presence of inhibitors were observed. These validation results verified that the SureID PanGlobal system provides robust genetic information suitable for forensic purposes representing a remarkable tool for forensic human identification and paternity testing. The use of SureID PanGlobal system would be also very beneficial to reduce the likelihood of adventitious matches as the number of profiles stored in the DNA database increases, and increase international compatibility to assist law enforcement data-sharing efforts.

## Electronic supplementary material

Supplementary element
