# Supplementary material for: Developmental validation of a 6-dye typing system with 27 loci and application in Han population of China
Source: Sci Rep. 2017 Jul 5;7:4706. doi: 10.1038/s41598-017-04548-1 (PMC5498568; doi:10.1038/s41598-017-04548-1)
Supplement: Supplementary file 1 — Supplementary element [file 41598_2017_4548_MOESM1_ESM.doc]

Developmental validation of a 6-dye typing system with 27 loci and application in Han population of China

Yaju Liu^1^, Lihong Guo^2^, Haiying Jin^3^ , Zheng Li^3^, Rufeng Bai^4^, Meisen Shi^4^, Shuhua Ma^5^


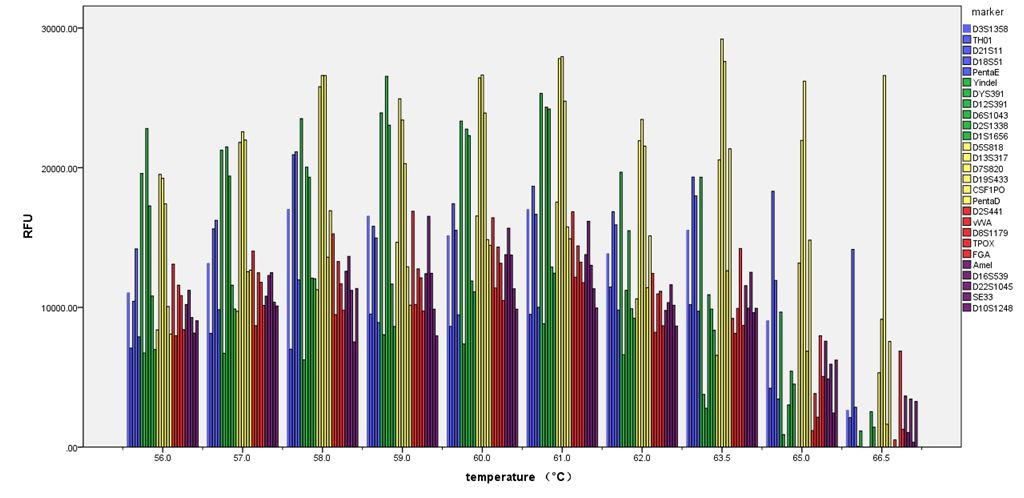


**Figure S1.** Representative electropherograms for reactions using annealing temperatures of 59-63.5°C are shown. Each bar represents an average peak height of duplicates. Bar colors represent the individual markers. The SureID PanGlobal system is based on the Applied Biosystems 3500XL Genetic Analyzers, with 36cm array and POP-4 polymer.


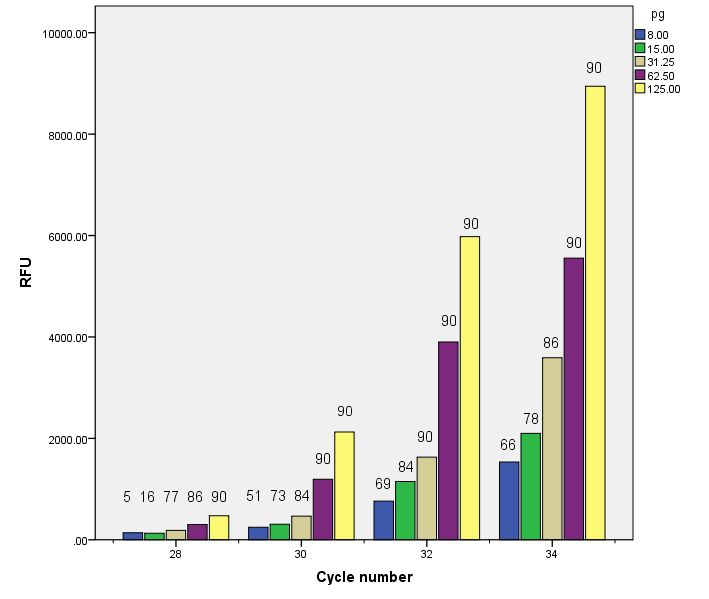


**Figure S2.** Positive Control 9948 (125, 62.5, 31.25,15 and 8 pg) was subjected to amplification using 28, 30, 32or 34 PCR cycles with the SureID PanGlobal system. The PCR products were analyzed using an Applied Biosystems 3500 Genetic Analyzer, with 36cm array and POP-4 polymer. Samples were run in duplicates. The numbers of detected PCR products (indicated above the bars; 90 PCR products expected) and their peak heights were indicated. 100 RFU was used as a threshold for detection.


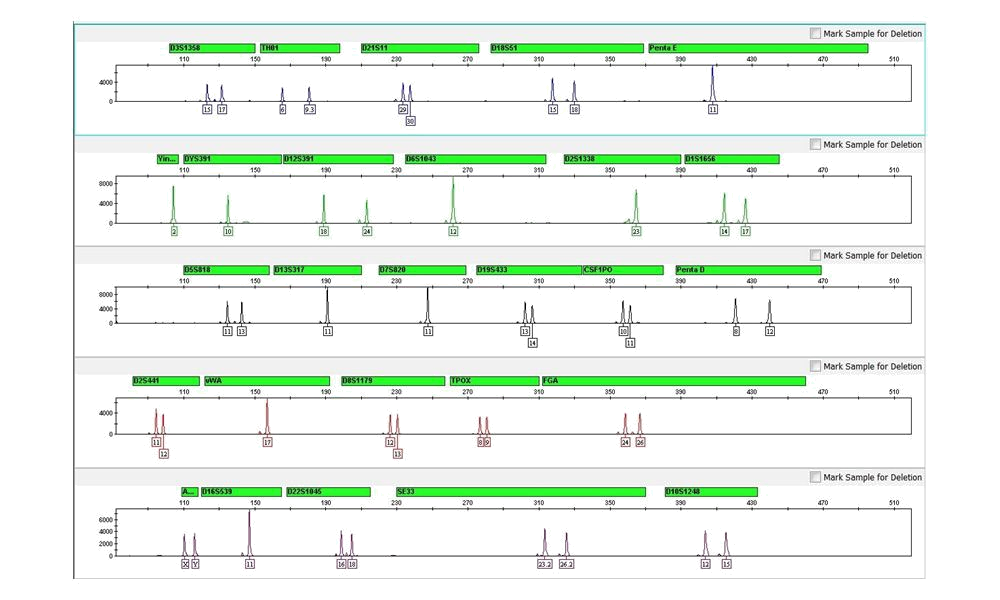


**Figure S3**. Electropherogram of control DNA 9948 (0.5 ng) amplified by the SureID PanGlobal system which based on the Applied Biosystems 3500XL Genetic Analyzers, 36cm array and POP-4 polymer.


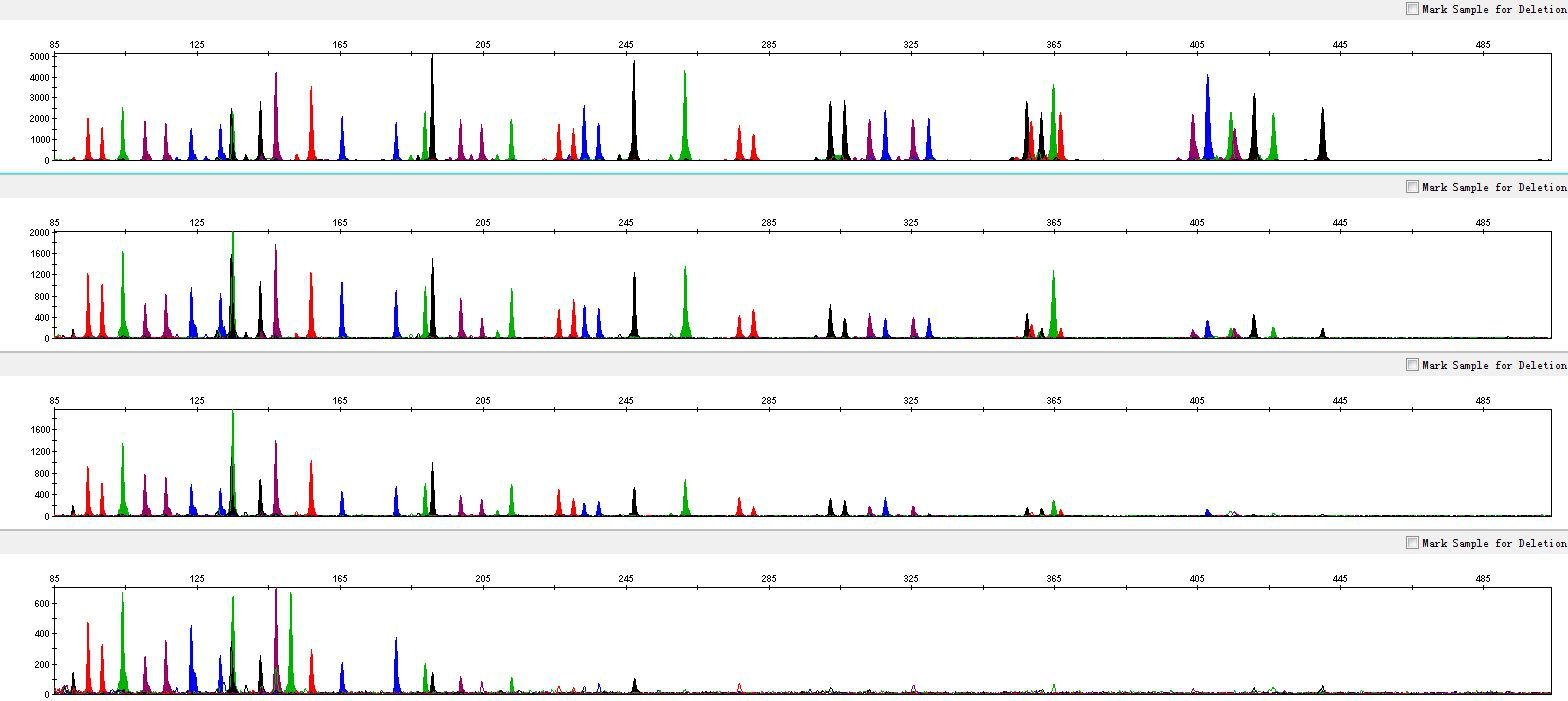


Control

500 bp

300 bp

150 bp

**Figure S4.** Control 9948 was sheared to the defined average fragments for STR analysis of untreated control and degraded DNA samples. 0.5 ng template DNA was used for control and degraded samples. Full profiles were obtained from samples of average fragment length of 500 bp.


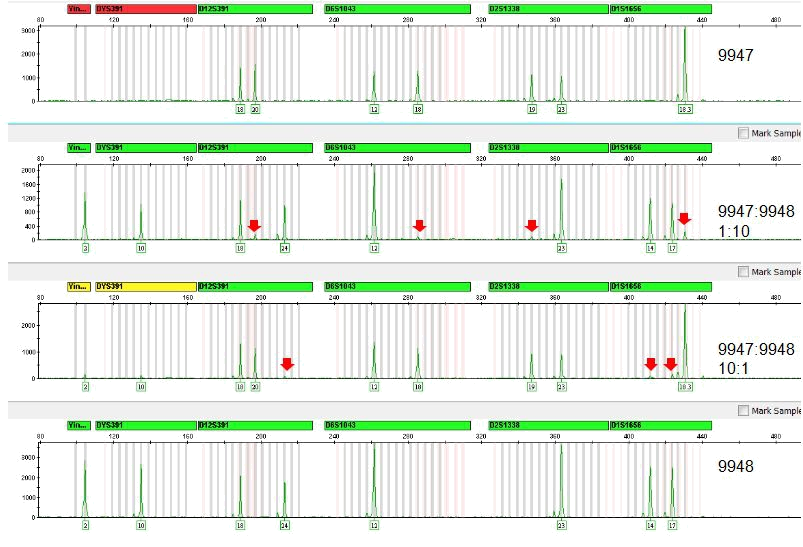


**Figure S5.** Results of the mixture analysis in the green channel. The figure shows the test results of 1:10 and 10:1 mixtures of DNA Control 9947 and 9948 (middle panels) and the corresponding single-source samples as a reference (upper and lower panel, respectively). Arrows indicate positions where a peak of the minor component DNA that does not overlap with a main peak, or a stutter peak of the major component, is to be expected. The tests are based on the Applied Biosystems 3500 XL Genetic Analyzers, 36cm array and POP-4 polymer.


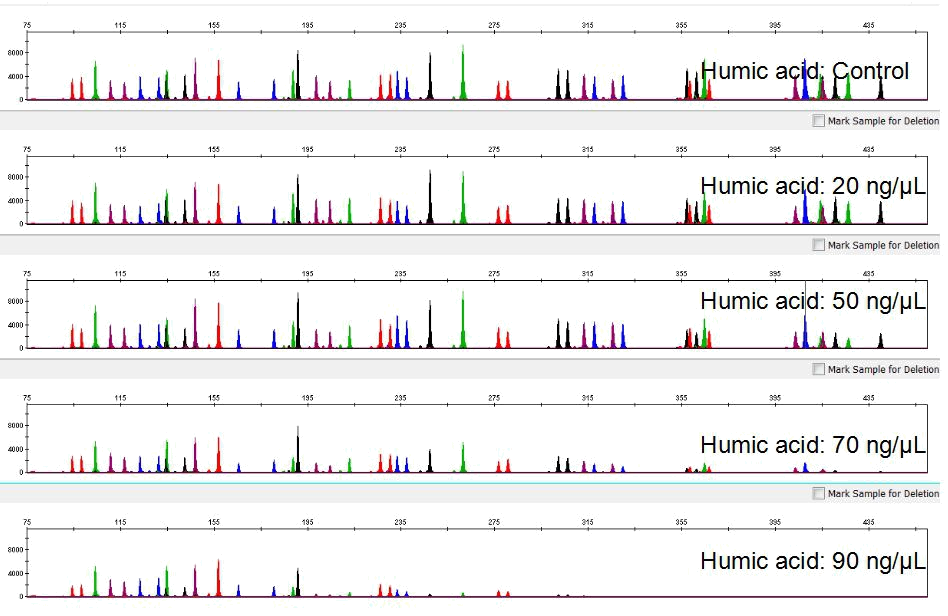


**Figure S6-1.** Positive control 9948 (0.5 ng) amplified with the SureID PanGlobal system in the presence of varying concentrations of humic acid and analyzed on Applied Biosystems 3500XL Genetic Analyzers, 36cm array and POP-4 polymer. Full profiles were obtained up to a concentration of humic acid of 70ng/μL.


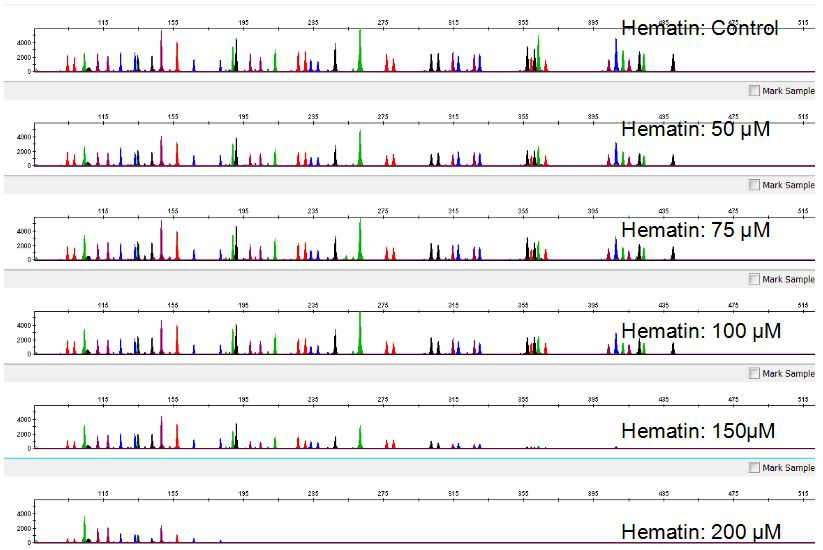


**Figure S6-2**.Positive control 9948 (0.5 ng) amplified with the SureID PanGlobal system in the presence of varying concentrations of Hematin and analyzed on Applied Biosystems 3500XL Genetic Analyzers, 36cm array and POP-4 polymer. Full profiles were obtained up to a concentration of hematin of 100 μM.


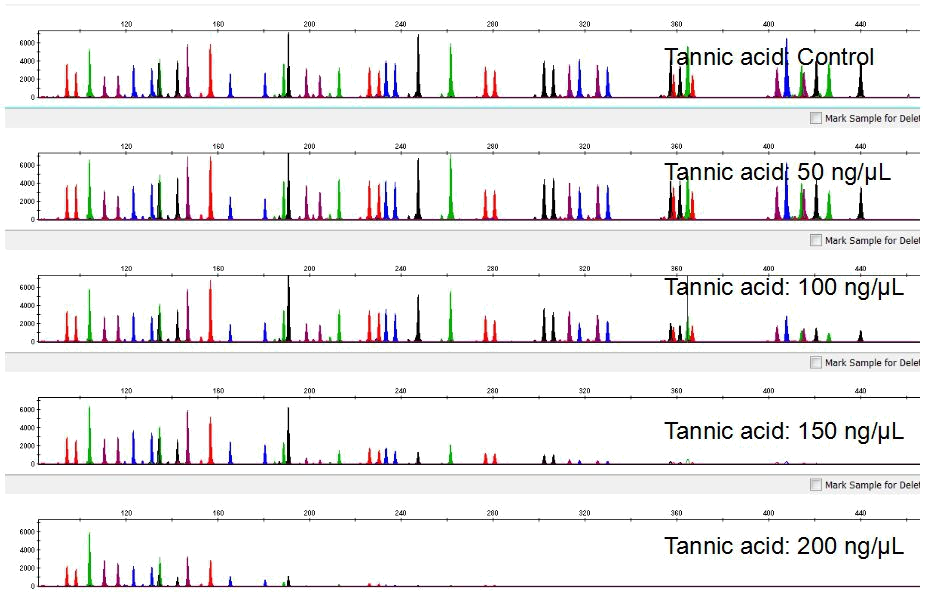


**Figure S6-3**.Positive control 9948 (0.5 ng) amplified with the SureID PanGlobal system in the presence of varying concentrations of tannic acid and analyzed on Applied Biosystems 3500XL Genetic Analyzers, 36cm array and POP-4 polymer. Full profiles were obtained up to a concentration of tannic acid of 100 ng/*μ*L.


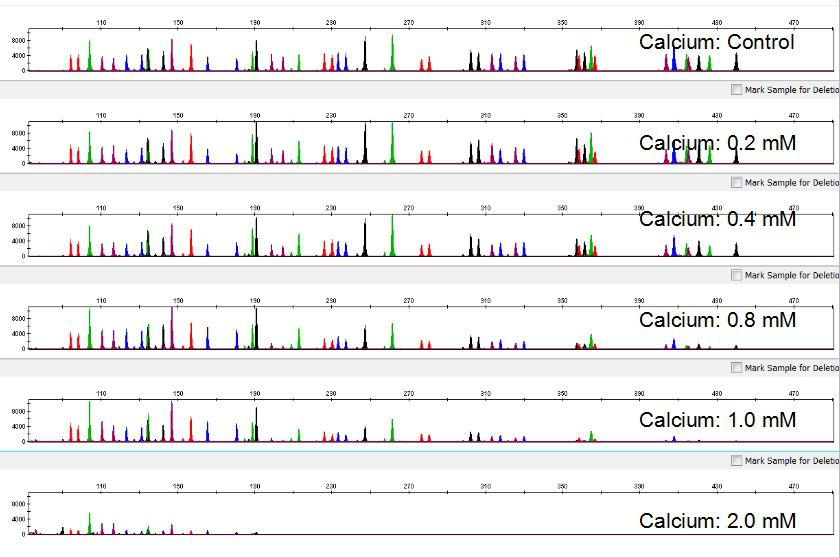


**Figure S6-4.**Positive control 9948 (0.5ng) amplified with the SureID PanGlobal system in the presence of varying concentrations of calcium chloride and analyzed on Applied Biosystems 3500XL Genetic Analyzers, 36cm array and POP-4 polymer. Full profiles were obtained up to a concentration of calcium chloride of 1.0 mM.


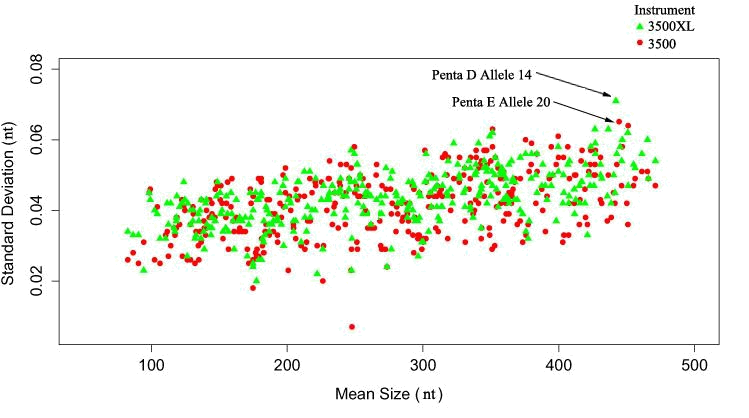


**Figure S7.** Size precision of allelic ladder alleles in SureID PanGlobal system. The average amplicon size of each allelic ladder allele was plotted against the standard deviation displayed from 240 samples (three successive runs) on the Applied Biosystems 3500 Genetic Analyzer (red circles) and 217 samples (three successive runs) on the Applied Biosystems 3500XL Genetic Analyzer (green triangles). Black arrows show the highest SD value on 3500 and 3500XL instruments.

| **Locus** | **Stutter mean(%)** | **Standard deviation(%)** | **Stutter filter** |
| --- | --- | --- | --- |
|  |  |  |  |
| D3S1358 | 9.88 | 2.36 | 16.94 |
|  |  |  |  |
| TH01 | 2.95 | 1.2 | 6.54 |
|  |  |  |  |
| D21S11 | 8.41 | 1.4 | 12.61 |
|  |  |  |  |
| D18S51 | 7.67 | 2.95 | 16.53 |
|  |  |  |  |
| Penta E | 4.73 | 2.04 | 10.84 |
|  |  |  |  |
| DYS391 | 6.11 | 1.12 | 9.46 |
|  |  |  |  |
| D12S391 | 10.79 | 4.01 | 22.82 |
|  |  |  |  |
| D6S1043 | 6.91 | 1.41 | 11.15 |
|  |  |  |  |
| D2S1338 | 9.63 | 2.03 | 15.71 |
|  |  |  |  |
| D1S1656 | 9.19 | 1.87 | 14.8 |
|  |  |  |  |
| D5S818 | 6.91 | 2.22 | 13.58 |
|  |  |  |  |
| D13S317 | 4.96 | 3.05 | 14.12 |
|  |  |  |  |
| D7S820 | 5.06 | 2.19 | 11.62 |
|  |  |  |  |
| D19S433 | 6.83 | 1.71 | 11.96 |
|  |  |  |  |
| CSFIPO | 6.29 | 1.85 | 11.84 |
|  |  |  |  |
| Penta D | 1.63 | 0.45 | 3.59 |
|  |  |  |  |
| D2S441 | 5.63 | 17.47 | 10.86 |
|  |  |  |  |

| vWA | 7.25 | 3.4 | 17.44 |
| --- | --- | --- | --- |
|  |  |  |  |
| D8S1179 | 7.08 | 0.74 | 9.3 |
|  |  |  |  |
| TPOX | 3.17 | 1.04 | 6.28 |
|  |  |  |  |
| FGA | 7.73 | 2.55 | 15.38 |
|  |  |  |  |
| D16S539 | 6.43 | 2.68 | 14.46 |
|  |  |  |  |
| D22S1045 | 8.11 | 4.01 | 20.14 |
|  |  |  |  |
| SE33 | 10.36 | 1.67 | 15.36 |
|  |  |  |  |
| D10S1248 | 8.11 | 1.43 | 12.42 |
|  |  |  |  |

**Table S1** Percent stutter for 25 STR loci from 457 genotyped population samples. A range of 125 pg-2 ng DNA templates was amplified for 30 cycles. Stutters were interpreted for samples with peak heights between 150 RFUs and 6000 RFUs. The analytical threshold of minimum stutter peak height was 20 RFUs. Stutter filter=Mean+3SD.


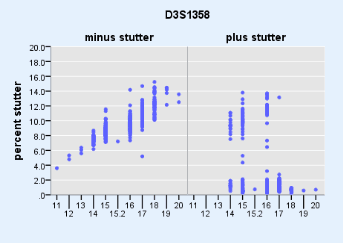

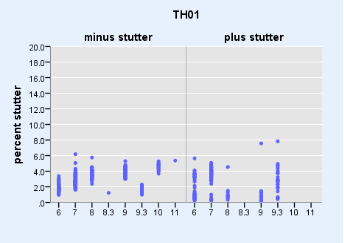


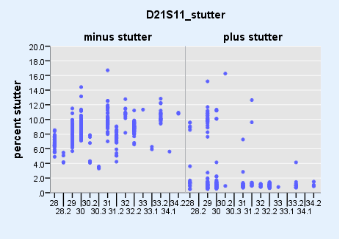

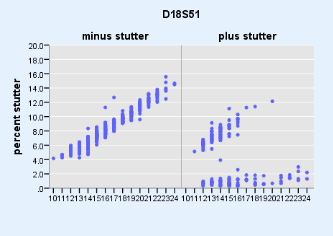


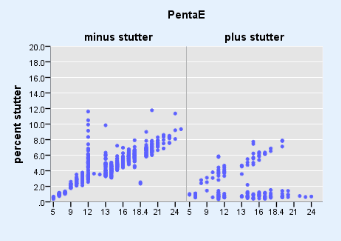

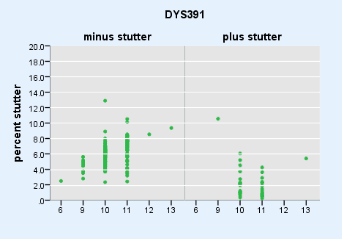


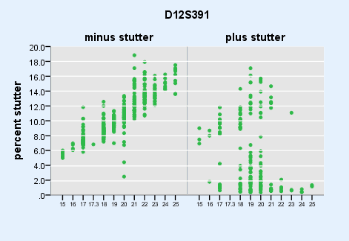

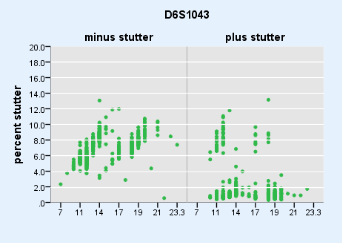


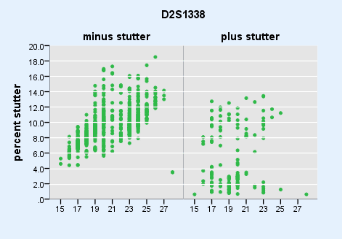

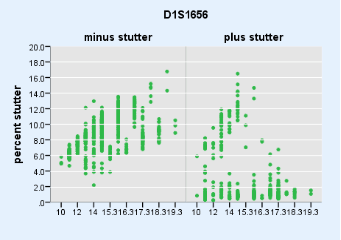


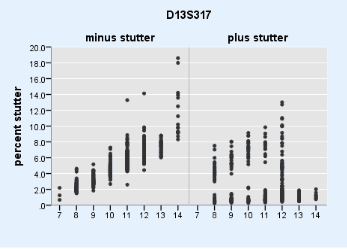


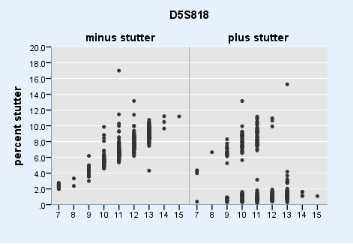


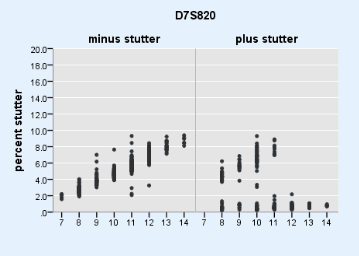

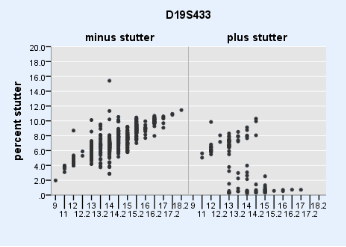


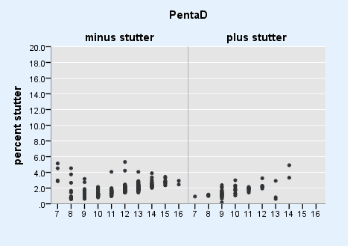

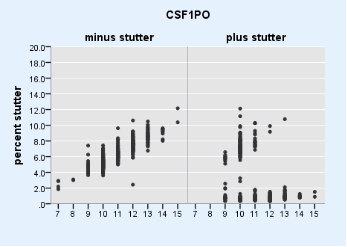


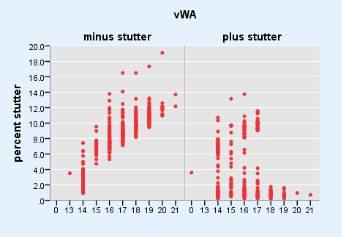

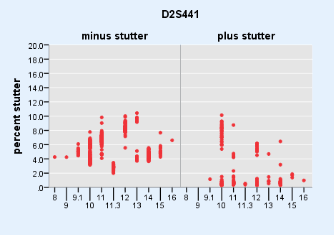


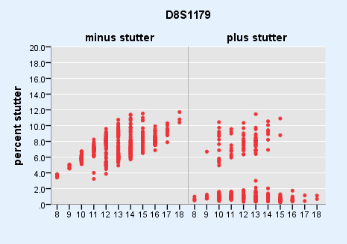

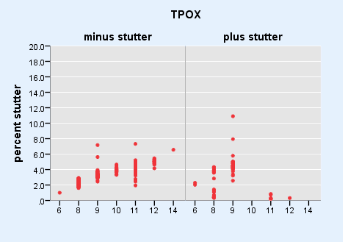


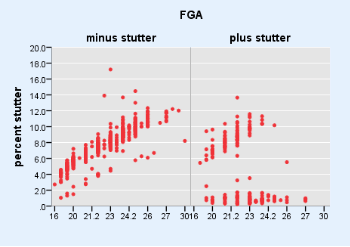

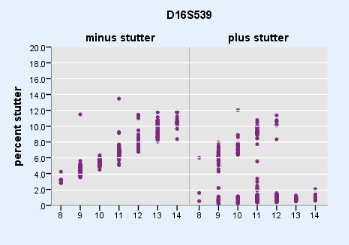


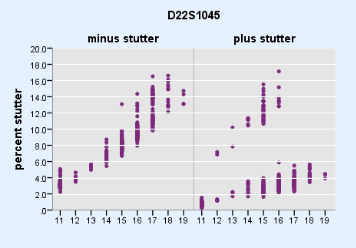

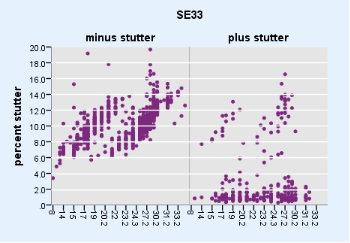


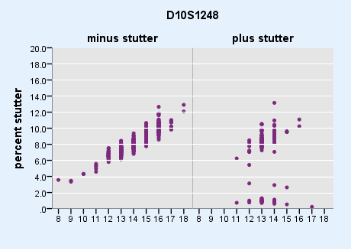


**Figure S8.** Stutter peak height ratios (above 25 pictures). Peak heights of minus and plus stutters compared to main peaks were analyzed for 457 DNA samples from different donors.


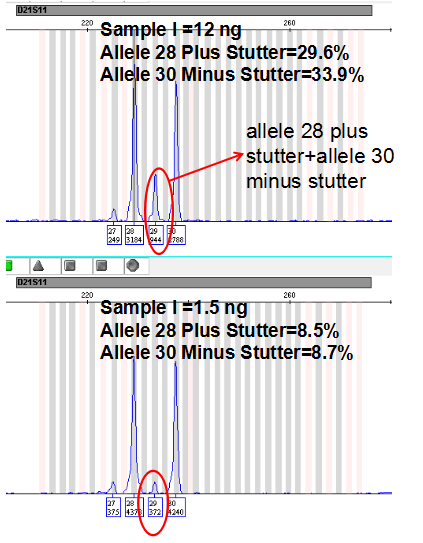

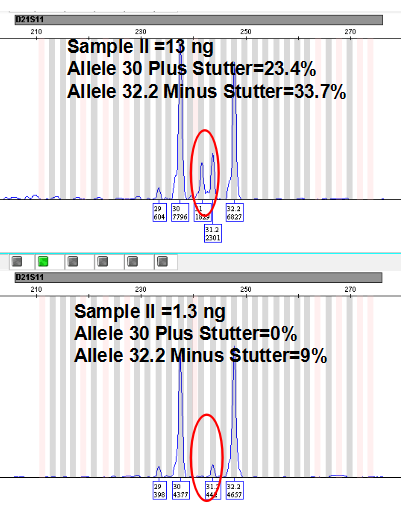


**Figure S9.** Stutter peak height ratios of D21S11 from two different donors with amplifications of different DNA concentrations. Lower the DNA concentration resulted in lower stutter peak height ratio of D21S11.

| 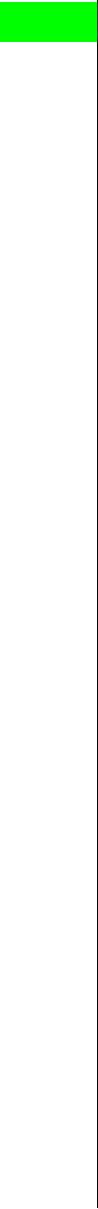Allele | D3S1358 | TH01 | | D21S11 | | D18S51 | | Penta | E |  | D12S39D6S1043 | | D2S1338 | D1S1656 | D5S818 |  | D13S317 | D7S820 | D19S433 |  | CSF1PO | PentaD | D2S441 | vWA | D8S1179 | TPOX | FGA | D16S539 | D22S1045 | SE33 | D10S1248 |
| --- | --- | --- | --- | --- | --- | --- | --- | --- | --- | --- | --- | --- | --- | --- | --- | --- | --- | --- | --- | --- | --- | --- | --- | --- | --- | --- | --- | --- | --- | --- | --- |
|  |  |  | |  | |  | |  | |  |  |  |  |  |  |  |  |  |  |  |  |  |  |  |  |  |  |  |  |  |  |
| 5 |  | 0.0004 | |  | |  | | 0.0502 | |  |  |  |  |  |  |  | 0.0009 |  |  |  |  |  |  |  |  |  |  |  |  |  |  |
|  |  |  | |  | |  | |  |  |  |  |  |  |  |  |  |  |  |  |  |  |  |  |  |  |  |  |  |  |  |  |
| 6 |  | 0.0876 | |  | |  | |  |  |  |  |  |  |  |  |  | 0.0004 |  |  |  |  | 0.0031 |  |  |  |  |  |  |  |  |  |
|  |  |  | |  | |  | |  | |  |  |  |  |  |  |  |  |  |  |  |  |  |  |  |  |  |  |  |  |  |  |
| 7 |  | 0.2632 | |  | | 0.0009 | | 0.0026 | |  |  |  |  |  | 0.0136 |  | 0.0013 | 0.0026 |  |  | 0.0013 | 0.0048 |  |  | 0.0004 | 0.0009 |  | 0.0004 |  |  |  |
|  |  |  | |  | |  | |  | |  |  |  |  |  |  |  |  |  |  |  |  |  |  |  |  |  |  |  |  |  |  |
| 8 |  | 0.0546 | |  | |  | | 0.0048 | |  |  |  |  |  | 0.0053 |  | 0.2557 | 0.1536 | 0.0004 |  | 0.0004 | 0.0519 |  |  | 0.0013 | 0.5119 |  | 0.0084 |  |  | 0.0013 |
|  |  |  | |  | |  | |  | |  |  |  |  |  |  |  |  |  |  |  |  |  |  |  |  |  |  |  |  |  |  |
| 9 |  | 0.5260 | |  | |  | | 0.0066 | |  |  | 0.0004 |  |  | 0.0581 |  | 0.1461 | 0.0555 |  |  | 0.0546 | 0.3143 | 0.0018 |  |  | 0.1210 |  | 0.2892 |  |  | 0.0004 |
|  |  |  | |  | |  | |  | |  |  |  |  |  |  |  |  |  |  |  |  |  |  |  |  |  |  |  |  |  |  |
| 10 |  | 0.0220 | |  | | 0.0004 | | 0.0379 | |  |  | 0.0321 |  | 0.0009 | 0.1897 |  | 0.1518 | 0.1668 |  |  | 0.2276 | 0.1061 | 0.2535 |  | 0.0911 | 0.0233 |  | 0.1127 |  |  | 0.0004 |
|  |  |  | |  | |  | |  | |  |  |  |  |  |  |  |  |  |  |  |  |  |  |  |  |  |  |  |  |  |  |
| 11 |  |  | |  | | 0.0026 | | 0.1219 | |  |  | 0.1065 |  | 0.0599 | 0.3261 |  | 0.2478 | 0.3411 | 0.0040 |  | 0.2394 | 0.1527 | 0.3539 |  | 0.0814 | 0.3055 |  | 0.2408 | 0.2469 |  | 0.0044 |
|  |  |  | |  | |  | |  | |  |  |  |  |  |  |  |  |  |  |  |  |  |  |  |  |  |  |  |  |  |  |
| 12 | 0.0022 |  | |  | | 0.0330 | | 0.1039 | |  |  | 0.1400 |  | 0.0387 | 0.2381 |  | 0.1518 | 0.2350 | 0.0365 |  | 0.3869 | 0.1972 | 0.1849 | 0.0004 | 0.1237 | 0.0339 |  | 0.2117 | 0.0048 | 0.0009 | 0.0885 |
|  |  |  | |  | |  | |  | |  |  |  |  |  |  |  |  |  |  |  |  |  |  |  |  |  |  |  |  |  |  |
| 13 | 0.0004 |  | |  | | 0.2196 | | 0.0515 | |  |  | 0.1329 |  | 0.1012 | 0.1598 |  | 0.0348 | 0.0383 | 0.2879 |  | 0.0814 | 0.1180 | 0.0233 | 0.0009 | 0.2438 | 0.0031 |  | 0.1162 | 0.0079 | 0.0044 | 0.3829 |
|  |  |  | |  | |  | |  | |  |  |  |  |  |  |  |  |  |  |  |  |  |  |  |  |  |  |  |  |  |  |
| 14 | 0.0396 |  | |  | | 0.2192 | | 0.0990 | |  |  | 0.1395 |  | 0.0616 | 0.0088 |  | 0.0084 | 0.0035 | 0.2575 |  | 0.0079 | 0.0423 | 0.1202 | 0.2372 | 0.2020 | 0.0004 | 0.0004 | 0.0189 | 0.0106 |  | 0.221 |
|  |  |  | |  | |  | |  | |  |  |  |  |  |  |  |  |  |  |  |  |  |  |  |  |  |  |  |  |  |  |
| 15 | 0.3473 |  | |  | | 0.1721 | | 0.1061 | |  | 0.0141 | 0.0132 |  | 0.3085 | 0.0004 |  | 0.0009 |  | 0.0660 |  | 0.0004 | 0.0088 | 0.0101 | 0.0326 | 0.1695 |  |  | 0.0018 | 0.2764 | 0.0123 | 0.1967 |
|  |  |  | |  | |  | |  | |  |  |  |  |  |  |  |  |  |  |  |  |  |  |  |  |  |  |  |  |  |  |
| 16 | 0.3433 |  | |  | | 0.1162 | | 0.0915 | |  | 0.0053 | 0.0035 | 0.0066 | 0.2333 |  |  |  |  | 0.0150 |  |  | 0.0009 | 0.0013 | 0.1840 | 0.0691 |  | 0.0009 |  | 0.2434 | 0.0255 | 0.0841 |
|  |  |  | |  | |  | |  | |  |  |  |  |  |  |  |  |  |  |  |  |  |  |  |  |  |  |  |  |  |  |
| 17 | 0.1954 |  | |  | | 0.0731 | | 0.0757 | |  | 0.1012 | 0.0361 | 0.0638 | 0.0876 |  |  |  |  |  |  |  |  |  | 0.2460 | 0.0136 |  | 0.0013 |  | 0.1871 | 0.0449 | 0.0189 |
|  |  |  | |  | |  | |  | |  |  |  |  |  |  |  |  |  |  |  |  |  |  |  |  |  |  |  |  |  |  |
| 18 | 0.0651 |  | |  | | 0.0427 | | 0.0744 | |  | 0.2460 | 0.1783 | 0.1193 | 0.0092 |  |  |  |  |  |  |  |  |  | 0.1875 | 0.0035 |  | 0.0238 |  | 0.0194 | 0.0607 | 0.0009 |
|  |  |  | |  | |  | |  | |  |  |  |  |  |  |  |  |  |  |  |  |  |  |  |  |  |  |  |  |  |  |
| 19 | 0.0057 |  | |  | | 0.0440 | | 0.0577 | |  | 0.2157 | 0.1430 | 0.1646 | 0.0009 |  |  |  |  |  |  |  |  |  | 0.0867 |  |  | 0.0370 |  | 0.0031 | 0.0713 | 0.0004 |
|  |  |  | |  | |  | |  | |  |  |  |  |  |  |  |  |  |  |  |  |  |  |  |  |  |  |  |  |  |  |
| 20 | 0.0009 |  | |  | | 0.0326 | | 0.0401 | |  | 0.1580 | 0.0581 | 0.1166 | 0.0004 |  |  |  |  |  |  |  |  |  | 0.0238 | 0.0004 |  | 0.0497 |  |  | 0.0691 |  |
|  |  |  | |  | |  | |  | |  |  |  |  |  |  |  |  |  |  |  |  |  |  |  |  |  |  |  |  |  |  |
| 21 |  |  | |  | | 0.0229 | | 0.0335 | |  | 0.1096 | 0.0088 | 0.0246 |  |  |  |  |  |  |  |  |  |  | 0.0009 |  |  | 0.1012 |  |  | 0.0599 |  |
|  |  |  | |  | |  | |  | |  |  |  |  |  |  |  |  |  |  |  |  |  |  |  |  |  |  |  |  |  |  |
| 22 |  |  | |  | | 0.0110 | | 0.0233 | |  | 0.0744 | 0.0009 | 0.0484 |  |  |  |  |  |  |  |  |  |  |  |  |  | 0.1730 |  |  | 0.0176 |  |
|  |  |  | |  | |  | |  | |  |  |  |  |  |  |  |  |  |  |  |  |  |  |  |  |  |  |  |  |  |  |
| 23 |  |  | |  | | 0.0048 | | 0.0123 | |  | 0.0409 |  | 0.2179 |  |  |  |  |  |  |  |  |  |  |  |  |  | 0.2271 |  |  | 0.0062 |  |
|  |  |  | |  | |  | |  | |  |  |  |  |  |  |  |  |  |  |  |  |  |  |  |  |  |  |  |  |  |  |
| 24 |  |  | |  | | 0.0035 | | 0.0044 | |  | 0.0198 |  | 0.1505 |  |  |  |  |  |  |  |  |  |  |  |  |  | 0.1915 |  |  | 0.0004 |  |
|  |  |  | |  | |  | |  | |  |  |  |  |  |  |  |  |  |  |  |  |  |  |  |  |  |  |  |  |  |  |
| 25 |  |  | |  | |  | | 0.0009 | |  | 0.0101 |  | 0.0700 |  |  |  |  |  |  |  |  |  |  |  |  |  | 0.0982 |  |  | 0.0004 |  |
|  |  |  | |  | |  | |  | |  |  |  |  |  |  |  |  |  |  |  |  |  |  |  |  |  |  |  |  |  |  |
| 26 |  |  | |  | | 0.0004 | | 0.0013 | |  | 0.0026 |  | 0.0150 |  |  |  |  |  |  |  |  |  |  |  |  |  | 0.0524 |  |  |  |  |
|  |  |  | |  | |  | |  | |  |  |  |  |  |  |  |  |  |  |  |  |  |  |  |  |  |  |  |  |  |  |
| 27 |  |  | | 0.0026 | | 0.0004 | | 0.0004 | |  |  |  | 0.0009 |  |  |  |  |  |  |  |  |  |  |  |  |  | 0.0092 |  |  | 0.0004 |  |
|  |  |  | |  | |  | |  |  |  |  |  |  |  |  |  |  |  |  |  |  |  |  |  |  |  |  |  |  |  |  |
| 28 |  |  | | 0.0440 | |  | |  |  |  |  |  | 0.0009 |  |  |  |  |  |  |  |  |  |  |  |  |  | 0.0013 |  |  | 0.0004 |  |
|  |  |  | |  | |  | |  |  |  |  |  |  |  |  |  |  |  |  |  |  |  |  |  |  |  |  |  |  |  |  |
|  | | |  | |  | |  | | | | | | | | | | | | | | | | | | | | | | | | |


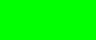


| 29 |  |  | 0.2716 |  |  |  |  | 0.0009 |  |  |  |  |  |  |  |  |  |  |  |  |  |  |  |  |
| --- | --- | --- | --- | --- | --- | --- | --- | --- | --- | --- | --- | --- | --- | --- | --- | --- | --- | --- | --- | --- | --- | --- | --- | --- |
|  |  |  |  |  |  |  |  |  |  |  |  |  |  |  |  |  |  |  |  |  |  |  |  |  |
| 30 |  |  | 0.2839 |  |  |  |  |  |  |  |  |  |  |  |  |  |  |  |  |  |  |  |  |  |
|  |  |  |  |  |  |  |  |  |  |  |  |  |  |  |  |  |  |  |  |  |  |  |  |  |
| 31 |  |  | 0.1012 |  |  |  |  |  |  |  |  |  |  |  |  |  |  |  |  |  |  |  |  |  |
|  |  |  |  |  |  |  |  |  |  |  |  |  |  |  |  |  |  |  |  |  |  |  |  |  |
| 32 |  |  | 0.0343 |  |  |  |  |  |  |  |  |  |  |  |  |  |  |  |  |  |  |  |  |  |
|  |  |  |  |  |  |  |  |  |  |  |  |  |  |  |  |  |  |  |  |  |  |  |  |  |
| 33 |  |  | 0.0022 |  |  |  |  |  |  |  |  |  |  |  |  |  |  |  |  |  |  |  |  |  |
|  |  |  |  |  |  |  |  |  |  |  |  |  |  |  |  |  |  |  |  |  |  |  |  |  |
| 34 |  |  | 0.0009 |  |  |  |  |  |  |  |  |  |  |  |  |  |  |  |  |  |  |  |  |  |
|  |  |  |  |  |  |  |  |  |  |  |  |  |  |  |  |  |  |  |  |  |  |  |  |  |
| 8.1 |  |  |  |  |  |  |  |  |  |  |  |  |  |  |  | 0.0004 |  |  |  |  |  |  |  |  |
|  |  |  |  |  |  |  |  |  |  |  |  |  |  |  |  |  |  |  |  |  |  |  |  |  |
| 9.1 |  |  |  |  |  |  |  |  |  |  |  | 0.0026 |  |  |  | 0.0198 |  |  |  |  |  |  |  |  |
|  |  |  |  |  |  |  |  |  |  |  |  |  |  |  |  |  |  |  |  |  |  |  |  |  |
| 9.3 |  | 0.0462 |  |  |  |  |  |  |  |  |  |  |  |  |  |  |  |  |  |  |  |  |  |  |
|  |  |  |  |  |  |  |  |  |  |  |  |  |  |  |  |  |  |  |  |  |  |  |  |  |
| 10.1 |  |  |  |  |  |  |  |  |  |  |  | 0.0009 |  |  |  | 0.0018 |  |  |  |  |  |  |  |  |
|  |  |  |  |  |  |  |  |  |  |  |  |  |  |  |  |  |  |  |  |  |  |  |  |  |
| 11.1 |  |  |  |  |  |  |  |  |  |  |  |  |  |  |  | 0.0004 |  |  |  |  |  |  |  |  |
|  |  |  |  |  |  |  |  |  |  |  |  |  |  |  |  |  |  |  |  |  |  |  |  |  |
| 11.3 |  |  |  |  |  |  |  |  |  |  |  |  |  |  |  | 0.0277 |  |  |  |  |  |  |  |  |
|  |  |  |  |  |  |  |  |  |  |  |  |  |  |  |  |  |  |  |  |  |  |  |  |  |
| 12.1 |  |  |  |  |  |  |  |  |  |  |  |  |  |  |  |  |  |  |  |  |  | 0.0004 | 0.0004 |  |
|  |  |  |  |  |  |  |  |  |  |  |  |  |  |  |  |  |  |  |  |  |  |  |  |  |
| 12.2 |  |  |  |  |  |  |  |  |  |  |  |  | 0.0079 |  |  |  |  |  |  |  |  |  |  |  |
|  |  |  |  |  |  |  |  |  |  |  |  |  |  |  |  |  |  |  |  |  |  |  |  |  |
| 12.3 |  |  |  |  |  |  |  |  |  |  |  |  |  |  |  | 0.0004 |  |  |  |  |  |  |  |  |
|  |  |  |  |  |  |  |  |  |  |  |  |  |  |  |  |  |  |  |  |  |  |  |  |  |
| 13.2 |  |  |  |  |  |  |  |  |  |  |  |  | 0.0405 |  |  |  |  |  |  |  |  |  | 0.0004 |  |
|  |  |  |  |  |  |  |  |  |  |  |  |  |  |  |  |  |  |  |  |  |  |  |  |  |
| 14.1 |  |  |  |  |  |  |  |  |  |  |  |  |  |  |  | 0.0004 |  |  |  |  |  |  | 0.0004 |  |
|  |  |  |  |  |  |  |  |  |  |  |  |  |  |  |  |  |  |  |  |  |  |  |  |  |
| 14.2 |  |  |  |  |  |  |  |  |  |  |  |  | 0.1026 |  |  |  |  |  |  |  |  |  | 0.0004 |  |
|  |  |  |  |  |  |  |  |  |  |  |  |  |  |  |  |  |  |  |  |  |  |  |  |  |
| 15.1 |  |  |  |  |  |  |  |  |  |  |  |  |  |  |  |  |  |  |  |  |  |  | 0.0022 |  |
|  |  |  |  |  |  |  |  |  |  |  |  |  |  |  |  |  |  |  |  |  |  |  |  |  |
| 15.2 |  |  |  |  |  |  |  |  |  |  |  |  | 0.1488 |  |  |  |  |  |  |  |  |  |  |  |
|  |  |  |  |  |  |  |  |  |  |  |  |  |  |  |  |  |  |  |  |  |  |  |  |  |
| 15.3 |  |  |  |  |  |  |  |  | 0.0035 |  |  |  |  |  |  |  |  |  |  |  |  |  |  |  |
|  |  |  |  |  |  |  |  |  |  |  |  |  |  |  |  |  |  |  |  |  |  |  |  |  |
| 16.1 |  |  |  | 0.0004 |  |  |  |  |  |  |  |  |  |  |  |  |  |  |  |  |  |  | 0.0009 |  |
|  |  |  |  |  |  |  |  |  |  |  |  |  |  |  |  |  |  |  |  |  |  |  |  |  |
| 16.2 |  |  |  |  |  |  |  |  |  |  |  |  | 0.0290 |  |  |  |  |  |  |  |  |  |  |  |
|  |  |  |  |  |  |  |  |  |  |  |  |  |  |  |  |  |  |  |  |  |  |  |  |  |
| 16.3 |  |  |  |  |  |  |  |  | 0.0057 |  |  |  |  |  |  |  |  |  |  |  |  |  |  |  |
|  |  |  |  |  |  |  |  |  |  |  |  |  |  |  |  |  |  |  |  |  |  |  |  |  |
| 17.1 |  |  |  |  |  |  |  |  |  |  |  |  |  |  |  |  |  |  |  |  |  |  | 0.0004 |  |
|  |  |  |  |  |  |  |  |  |  |  |  |  |  |  |  |  |  |  |  |  |  |  |  |  |

| 17.2 |  |  |  |  |  |  |  |  |  |  |  |  | 0.0035 |  |  |  |  |  |  |  |  |  |  |  |
| --- | --- | --- | --- | --- | --- | --- | --- | --- | --- | --- | --- | --- | --- | --- | --- | --- | --- | --- | --- | --- | --- | --- | --- | --- |
|  |  |  |  |  |  |  |  |  |  |  |  |  |  |  |  |  |  |  |  |  |  |  |  |  |
| 17.3 |  |  |  |  |  | 0.0009 |  |  | 0.0607 |  |  |  |  |  |  |  |  |  |  |  |  |  |  |  |
|  |  |  |  |  |  |  |  |  |  |  |  |  |  |  |  |  |  |  |  |  |  |  |  |  |
| 18.2 |  |  |  |  |  | 0.0004 |  |  |  |  |  |  | 0.0004 |  |  |  |  |  |  |  |  |  | 0.0009 |  |
|  |  |  |  |  |  |  |  |  |  |  |  |  |  |  |  |  |  |  |  |  |  |  |  |  |
| 18.3 |  |  |  |  |  | 0.0004 |  |  | 0.0233 |  |  |  |  |  |  |  |  |  |  |  |  |  |  |  |
|  |  |  |  |  |  |  |  |  |  |  |  |  |  |  |  |  |  |  |  |  |  |  |  |  |
| 19.2 |  |  |  |  |  |  |  |  |  |  |  |  |  |  |  |  |  |  |  | 0.0004 |  |  | 0.0013 |  |
|  |  |  |  |  |  |  |  |  |  |  |  |  |  |  |  |  |  |  |  |  |  |  |  |  |
| 19.3 |  |  |  |  |  | 0.0004 |  |  | 0.0040 |  |  |  |  |  |  |  |  |  |  |  |  |  |  |  |
|  |  |  |  |  |  |  |  |  |  |  |  |  |  |  |  |  |  |  |  |  |  |  |  |  |
| 20.2 |  |  |  |  |  |  |  |  |  |  |  |  |  |  |  |  |  |  |  | 0.0004 |  |  | 0.0066 |  |
|  |  |  |  |  |  |  |  |  |  |  |  |  |  |  |  |  |  |  |  |  |  |  |  |  |
| 20.3 |  |  |  |  |  |  | 0.0022 |  | 0.0004 |  |  |  |  |  |  |  |  |  |  |  |  |  |  |  |
|  |  |  |  |  |  |  |  |  |  |  |  |  |  |  |  |  |  |  |  |  |  |  |  |  |
| 21.1 |  |  |  |  |  |  |  |  |  |  |  |  |  |  |  |  |  |  |  |  |  |  | 0.0009 |  |
|  |  |  |  |  |  |  |  |  |  |  |  |  |  |  |  |  |  |  |  |  |  |  |  |  |
| 21.2 |  |  |  |  |  |  |  |  |  |  |  |  |  |  |  |  |  |  |  | 0.0031 |  |  | 0.0172 |  |
|  |  |  |  |  |  |  |  |  |  |  |  |  |  |  |  |  |  |  |  |  |  |  |  |  |
| 21.3 |  |  |  |  |  |  | 0.0026 |  |  |  |  |  |  |  |  |  |  |  |  |  |  |  |  |  |
|  |  |  |  |  |  |  |  |  |  |  |  |  |  |  |  |  |  |  |  |  |  |  |  |  |
| 22.2 |  |  |  |  |  |  |  |  |  |  |  |  |  |  |  |  |  |  |  | 0.0084 |  |  | 0.0286 |  |
|  |  |  |  |  |  |  |  |  |  |  |  |  |  |  |  |  |  |  |  |  |  |  |  |  |
| 23.2 |  |  |  |  |  |  |  |  |  |  |  |  |  |  |  |  |  |  |  | 0.0101 |  |  | 0.0409 |  |
|  |  |  |  |  |  |  |  |  |  |  |  |  |  |  |  |  |  |  |  |  |  |  |  |  |
| 23.3 |  |  |  |  |  |  | 0.0018 |  |  |  |  |  |  |  |  |  |  |  |  |  |  |  | 0.0004 |  |
|  |  |  |  |  |  |  |  |  |  |  |  |  |  |  |  |  |  |  |  |  |  |  |  |  |
| 24.2 |  |  |  |  |  |  |  |  |  |  |  |  |  |  |  |  |  |  |  | 0.0070 |  |  | 0.0678 |  |
|  |  |  |  |  |  |  |  |  |  |  |  |  |  |  |  |  |  |  |  |  |  |  |  |  |
| 25.2 |  |  |  |  |  |  |  |  |  |  |  |  |  |  |  |  |  |  |  | 0.0022 |  |  | 0.0753 |  |
|  |  |  |  |  |  |  |  |  |  |  |  |  |  |  |  |  |  |  |  |  |  |  |  |  |
| 26.2 |  |  |  |  |  |  |  |  |  |  |  |  |  |  |  |  |  |  |  | 0.0009 |  |  | 0.0748 |  |
|  |  |  |  |  |  |  |  |  |  |  |  |  |  |  |  |  |  |  |  |  |  |  |  |  |
| 27.1 |  |  |  |  |  |  |  |  |  |  |  |  |  |  |  |  |  |  |  |  |  |  | 0.0004 |  |
|  |  |  |  |  |  |  |  |  |  |  |  |  |  |  |  |  |  |  |  |  |  |  |  |  |
| 27.2 |  |  |  |  |  |  |  |  |  |  |  |  |  |  |  |  |  |  |  | 0.0004 |  |  | 0.0717 |  |
|  |  |  |  |  |  |  |  |  |  |  |  |  |  |  |  |  |  |  |  |  |  |  |  |  |
| 28.2 |  |  | 0.0114 |  |  |  |  |  |  |  |  |  |  |  |  |  |  |  |  |  |  |  | 0.0775 |  |
|  |  |  |  |  |  |  |  |  |  |  |  |  |  |  |  |  |  |  |  |  |  |  |  |  |
| 28.3 |  |  | 0.0004 |  |  |  |  |  |  |  |  |  |  |  |  |  |  |  |  |  |  |  | 0.0004 |  |
|  |  |  |  |  |  |  |  |  |  |  |  |  |  |  |  |  |  |  |  |  |  |  |  |  |
| 29.2 |  |  | 0.0013 |  |  |  |  |  |  |  |  |  |  |  |  |  |  |  |  |  |  |  | 0.0612 |  |
|  |  |  |  |  |  |  |  |  |  |  |  |  |  |  |  |  |  |  |  |  |  |  |  |  |
| 30.2 |  |  | 0.0106 |  |  |  |  |  |  |  |  |  |  |  |  |  |  |  |  |  |  |  | 0.0409 |  |
|  |  |  |  |  |  |  |  |  |  |  |  |  |  |  |  |  |  |  |  |  |  |  |  |  |
| 30.3 |  |  | 0.0048 |  |  |  |  |  |  |  |  |  |  |  |  |  |  |  |  |  |  |  |  |  |
|  |  |  |  |  |  |  |  |  |  |  |  |  |  |  |  |  |  |  |  |  |  |  |  |  |
| 31.2 |  |  | 0.0709 |  |  |  |  |  |  |  |  |  |  |  |  |  |  |  |  |  |  |  | 0.0277 |  |
|  |  |  |  |  |  |  |  |  |  |  |  |  |  |  |  |  |  |  |  |  |  |  |  |  |

| 32.2 |  |  | 0.1114 |  |  |  |  |  |  |  |  |  |  |  |  |  |  |  |  |  |  |  |  |  |  |  |  |  |  | 0.0141 |  |
| --- | --- | --- | --- | --- | --- | --- | --- | --- | --- | --- | --- | --- | --- | --- | --- | --- | --- | --- | --- | --- | --- | --- | --- | --- | --- | --- | --- | --- | --- | --- | --- |
|  |  |  |  |  |  |  |  |  |  |  |  |  |  |  |  |  |  |  |  |  |  |  |  |  |  |  |  |  |  |  |  |
| 33.2 |  |  | 0.0445 |  |  |  |  |  |  |  |  |  |  |  |  |  |  |  |  |  |  |  |  |  |  |  |  |  |  | 0.0048 |  |
|  |  |  |  |  |  |  |  |  |  |  |  |  |  |  |  |  |  |  |  |  |  |  |  |  |  |  |  |  |  |  |  |
| 34.2 |  |  | 0.0035 |  |  |  |  |  |  |  |  |  |  |  |  |  |  |  |  |  |  |  |  |  |  |  |  |  |  | 0.0022 |  |
|  |  |  |  |  |  |  |  |  |  |  |  |  |  |  |  |  |  |  |  |  |  |  |  |  |  |  |  |  |  |  |  |
| 35.2 |  |  | 0.0004 |  |  |  |  |  |  |  |  |  |  |  |  |  |  |  |  |  |  |  |  |  |  |  |  |  |  | 0.0009 |  |
|  |  |  |  |  |  |  |  |  |  |  |  |  |  |  |  |  |  |  |  |  |  |  |  |  |  |  |  |  |  |  |  |
| N | 9 | 7 | 18 | 19 |  | 22 | 16 | 17 |  | 14 |  | 17 | 9 |  | 11 | 10 |  | 14 | 9 | 11 | 15 | 10 |  | 12 | 8 |  | 23 | 9 | 10 | 45 | 12 |
|  |  |  |  |  |  |  |  |  |  |  |  |  |  |  |  |  |  |  |  |  |  |  |  |  |  |  |  |  |  |  |  |
| He | 0.7148 | 0.6347 | 0.8081 | 0.8548 |  | 0.9210 | 0.8451 | 0.8740 |  | 0.8741 |  | 0.8213 | 0.7817 |  | 0.7782 | 0.7870 |  | 0.8019 | 0.7438 | 0.7960 | 0.7641 | 0.8116 |  | 0.8283 | 0.6303 |  | 0.8530 | 0.7835 | 0.7658 | 0.9357 | 0.7632 |
|  |  |  |  |  |  |  |  |  |  |  |  |  |  |  |  |  |  |  |  |  |  |  |  |  |  |  |  |  |  |  |  |
| DP | 0.8681 | 0.8264 | 0.9422 | 0.9582 |  | 0.9870 | 0.9531 | 0.9700 |  | 0.9655 |  | 0.9451 | 0.9120 |  | 0.9351 | 0.9115 |  | 0.9387 | 0.8804 | 0.9410 | 0.9062 | 0.9330 |  | 0.9532 | 0.8040 |  | 0.9619 | 0.9226 | 0.9062 | 0.9930 | 0.8969 |
|  |  |  |  |  |  |  |  |  |  |  |  |  |  |  |  |  |  |  |  |  |  |  |  |  |  |  |  |  |  |  |  |
| PIC | 0.6676 | 0.5932 | 0.7897 | 0.8315 |  | 0.9100 | 0.8182 | 0.8600 |  | 0.8479 |  | 0.7987 | 0.7361 |  | 0.7758 | 0.7385 |  | 0.7854 | 0.6879 | 0.7900 | 0.7237 | 0.7763 |  | 0.8152 | 0.5677 |  | 0.8384 | 0.7543 | 0.7282 | 0.9404 | 0.7138 |
|  |  |  |  |  |  |  |  |  |  |  |  |  |  |  |  |  |  |  |  |  |  |  |  |  |  |  |  |  |  |  |  |
| PEtrio | 0.4714 | 0.4039 | 0.6409 | 0.7005 |  | 0.8380 | 0.6789 | 0.7430 |  | 0.7249 |  | 0.6555 | 0.5577 |  | 0.6116 | 0.5633 |  | 0.6329 | 0.4973 | 0.5910 | 0.5449 | 0.6125 |  | 0.6727 | 0.3698 |  | 0.7113 | 0.5817 | 0.5425 | 0.8846 | 0.5330 |
|  |  |  |  |  |  |  |  |  |  |  |  |  |  |  |  |  |  |  |  |  |  |  |  |  |  |  |  |  |  |  |  |
| PEduo | 0.3004 | 0.2358 | 0.4674 | 0.5367 |  | 0.6723 | 0.5102 | 0.5840 |  | 0.5658 |  | 0.4834 | 0.3789 |  | 0.4340 | 0.3840 |  | 0.4584 | 0.3214 | 0.4060 | 0.3665 | 0.4355 |  | 0.5022 | 0.2146 |  | 0.5496 | 0.4031 | 0.3648 | 0.7930 | 0.3535 |
|  |  |  |  |  |  |  |  |  |  |  |  |  |  |  |  |  |  |  |  |  |  |  |  |  |  |  |  |  |  |  |  |
| *P* | 0.8206 | 0.6525 | 0.6743 | 0.5904 |  | 0.9831 | 0.5269 | 0.9972 |  | 0.2813 |  | 0.8790 | 0.4405 |  | 0.0635 | 0.2527 |  | 0.4852 | 0.3621 | 0.2343 | 0.7716 | 0.5958 |  | 0.4756 | 0.9063 |  | 0.8582 | 0.7504 | 0.8539 | 0.2369 | 0.3376 |
|  |  |  |  |  |  |  |  |  |  |  |  |  |  |  |  |  |  |  |  |  |  |  |  |  |  |  |  |  |  |  |  |

**Table S2** Allele frequencies and genetic parameters for 24 autosomal STR loci in Central Chinese Han population(n=1136). N:Number of alleles; He: Heterozygosity; DP: Discrimination power; PIC: Polymorphism information content; PEtrio: Probability of exclusion for trios; PEduo: Probability of exclusion of duos; P: probability values of exact tests for Hardy-Weinberg equilibrium (HWE).
